# The Method of Data Analysis in Intuitionistic Fuzzy Generalized Consistent Decision Formal Context

**DOI:** 10.3390/e21030262

**Published:** 2019-03-07

**Authors:** Binbin Sang, Binghan Long, Jinzhong Pang, Weihua Xu

**Affiliations:** 1 School of Sciences, Chongqing University of Technology, Chongqing 400054, China; 2 School of Mathematics and Statistics, Southwest University, Chongqing 400715, China

**Keywords:** attribute reduction, concept lattice, discernibility matrix, generalized consistent decision formal context, intuitionistic fuzzy set

## Abstract

Concept lattice has been successfully applied to various fields as an effective tool for data analysis and knowledge discovery, with attribute reduction being the key problem. This paper combines the intuitionistic fuzzy theory with the concept lattice theory and proposes one kind of concept lattice in intuitionistic fuzzy generalized consistent decision formal context. Furthermore, an approach to attribute a reduction in the discernibility matrix is proposed and investigated, making the discovery of implicit knowledge easier and the representation simpler in the data system and perfecting the theory of concept lattice. Moreover, this paper studies, in detail, the algorithms and case study of data analysis in the intuitionistic fuzzy generalized consistent decision formal context. The potential value of the method to deal with information discussed in this paper, especially the value of forecasting and decision-making, is expected in future.

## 1. Introduction

Concept lattice, which is also called Galois lattice, stems from the so-called formal concept analysis that was proposed by Wille in 1982 [1], which is oriented towards the discovery and design of concept hierarchies from relational databases. A classical concept lattice is defined by a binary relation between a set of objects and a set of attributes. Many researches on concept lattice focus on the theories, such as construction of concept lattice and fuzzy concept lattice [2,3,4,5], approach to pruning of concept lattice [6,7,8,9,10,11,12,13,14,15,16,17,18,19,20], acquisition of rules [10,20,21,22,23], relationship with rough set [24,25,26,27,28,29], and so they have much in common in terms of goals and methodologies. Although rough set theory [5,30] and formal context analysis are different theories, the combination with granular computing [31,32] and the paper [33,34,35,36] introduced a possibility-theoretic view of formal concept analysis, the construction of the characteristic matrixes of the dynamic coverings and a cognitive system model was established in formal concept analysis. The paper [37] was concerned with the relationship between contexts, closure spaces, and complete lattices. As an effective tool for data analysis and knowledge processing, concept lattice has been applied to various fields, such as data mining, software engineering, information retrieval, and so on [38,39,40,41,42].

The concept of intuitionistic fuzzy (IF, for short) set theory that was introduced by Atanassov [43,44,45,46] is also an important mathematical structure to cope with imprecise information. The IF set, which is a generalization of the fuzzy set of Zadeh’s fuzzy set [47], considers both membership degree and non-membership degree that are functions valued in [0,1], while a fuzzy set gives a membership degree only. The IF set, inducing an indeterminacy index, provide us with the possibility to model hesitation and uncertainty as to how an object satisfies a particular property by using an additional parameter, which is called the hesitation degree or the intuitionistic fuzzy index. Accordingly, in comparison with the fuzzy set, the IF set theory can present vague information better. In fact, IF set theory has been successfully applied in decision analysis and pattern recognition [48,49,50,51]. 

In paper [52], the discernibility matrix was proposed by using of the cuts of IF sets and then established the method of the attribute reduction of IF concept lattice, but for the requirement of knowledge handling systems, combining IF set theory and formal concept analysis theory may result in a new hybrid mathematical structure from other views, such as [53]. The knowledge reduction that was analyzed in the IF consistent decision formal context is usually obtained in the equivalence relation between conditional attribute concepts and decision attribute concepts. In fact, it only needs to satisfy the implication relation when dealing with data in real life. Therefore, it is necessary to determine the implication relationship between the concept of conditional attributes and the concept of decision attributes. 

In the real world, many cases request that there are correlations between different decision-making layers, which is to say that the correlation should exist between the concepts obtained in $\left(U,A,\tilde{I},\right)$ and those obtained in $\left(U,T,\tilde{J}\right)$. In the paper, the implication relation is applied.

In this paper, we aim to study attribute reduction in concept lattices in IF generalized consistent decision formal context. Attribute reduction is to find the minimum conditional attribute set that can keep the generalized consistence of the IF decision formal context. Not only does it make the discovery of implicit knowledge easier and the representation simpler in data, but it also extends the theory of concept lattice. Combing the IF theory and formal concept analysis, the data tables, in which the implication relation is introduced to structure a pair of Galois operators and establishe related concept lattice, is actually significant in decision formal concept analysis. Furthermore, the notion of coarser is generalized, which ascertains the implication relation between the conditional attribute concept and the decision attribute concept. Subsequently, some important propositions and conclusions are obtained regarding the attribute reduction in IF generalized consistent decision formal context. In this paper, the discernibility matrix is applied to the reduction of IF generalized consistent decision formal context. The reduction method can get all of the reduction of IF generalized consistent decision formal context. However, sometimes we do not need all reductions, and this method cannot get one of them quickly instead of all reductions.

The paper is organized, as follows. Section 2 reviews basic definitions in the formal concept analysis and IF formal decision concept analysis. In Section 3, we give the definitions and propositions of IF generalized consistent decision formal context and discuss the approach towards attribute reduction in concept lattices in IF generalized consistent decision formal context in the implication mapping f, and then we divide the attributes into four types as well as investigate some propositions to determine the type of an attribute. Furthermore, the discernibility matrix and discernibility function are introduced to compute all of the reductions based on implication mapping f. In next section, the attribute reduction of IF generalized consistent decision formal context is introduced and then we investigate the relation with the reduction based on the implication mappings. In fact, an approach to compute all reductions of IF generalized consistent decision formal context is obtained. In Section 5, we show the corresponding reduction algorithm. Finally, a simple conclusion is given in the paper.

## 2. Preliminaries

Firstly, to make this paper self-contained, IF set theory, the involved notions of formal concept analysis are briefly introduced and then the notions of IF formal concept analysis and IF decision formal concept analysis are reviewed. A detailed description of them can be found in corresponding references.

**Definition** **1.** *(Ganter and Wille [39]) A triple*(U,A,I)*is called a formal context, if*$U=\{x_{1},x_{2},\cdots ,x_{n}\}$*is an object set, where*$x_{i}(i\leq n)$*is called an object,*$A=\{a_{1},a_{2},\cdots ,a_{m}\}$*is an attribute set, where*$a_{j}(j\leq m)$*is called an attribute, and*I⊆U×A*is a binary relation between*U*and*A.

In a formal context (U,A,I), if (x,a)∈I i.e., xIa, we say that the object x has the attribute a, or that a is fulfilled by x. For convenience, we use “1” and “0” to represent (x,a)∈I and (x,a)∉I respectively. Thus, a table with only 0 and 1 can represent a formal context.

For a formal context (U,A,I), a pair of dual operators for X⊆U and B⊆A are defined in the following.

$$X^{*}=\{a\in A|(x,a)\in I,\forall x\in X\},$$

$$B^{*}=\{x\in U|(x,a)\in I,\forall a\in B\}.$$

In fact, $X^{*}$ is the set of all the attributes shared by all the objects in X, and $B^{*}$ is the set of all the objects that fulfill all of the attributes in B.

Meanwhile, the complement set of $X^{*}$ and $B^{*}$ are denoted by $\sim X^{*}$ and $\sim B^{*}$ where $\sim X^{*}=\{a\in A|(x,a)\notin I,\exists x\in X\}$ and $\sim B^{*}=\{x\in U|(x,a)\notin I,\exists a\in B\}.$

**Proposition** **1.** *(Ganter and Wille [39]) Let*(U,A,I)*be a formal context,*$X_{1},X_{2},X\subseteq U$*and*$B_{1},B_{2},B\subseteq A$, *the following properties hold.*

(1) $X_{1}\subseteq X_{2}\Rightarrow X_{2}^{*}\subseteq X_{1}^{*},B_{1}\subseteq B_{2}\Rightarrow B_{2}^{*}\subseteq B_{1}^{*}.$
(2) $X\subseteq X^{**},B\subseteq B^{**}.$
(3) $X^{*}=X^{***},B^{*}=B^{***}.$
(4) $X\subseteq B^{*}\Leftrightarrow B\subseteq X^{*}.$
(5) ${\left(X_{1}\cup X_{2}\right)}^{*}=X_{1}^{*}\cap X_{2}^{*},{\left(B_{1}\cup B_{2}\right)}^{*}=B_{1}^{*}\cap B_{2}^{*}.$
(6) ${\left(X_{1}\cap X_{2}\right)}^{*}⊇X_{1}^{*}\cup X_{2}^{*},{\left(B_{1}\cap B_{2}\right)}^{*}⊇B_{1}^{*}\cup B_{2}^{*}.$

**Definition** **2.** *(Ganter and Wille [39]) Let* (U,A,I) *be a formal context. A pair* (X,B) *is called a formal concept (in brief a concept) if* $X^{*}=B$ *and* $X=B^{*}$ *for* X⊆U,B⊆A *. Furthermore,* X *and* B *are called the extension and the intension of* (X,B) *, respectively.*

From the above discussions, it is clear that both $\left(X^{**},X^{*}\right)$ and $\left(B^{*},B^{**}\right)$ are concepts.

According to the references [6] and [45], we have the corresponding account, as follows.

For convenience, all concepts of a formal context (U,A,I) are denoted by L(U,A,I), and they are ordered by:

$$(X_{1},B_{1})\leq (X_{2},B_{2})\Leftrightarrow X_{1}\subseteq X_{2}\Leftrightarrow B_{1}⊇B_{2}$$

where $\left(X_{1},B_{1}\right)$ and $\left(X_{2},B_{2}\right)$ are concepts. Moreover, $\left(X_{1},B_{1}\right)$ is called a sub-concept of $\left(X_{2},B_{2}\right)$, and $\left(X_{2},B_{2}\right)$ is called a super-concept of $\left(X_{1},B_{1}\right)$. $\left(X_{1},B_{1})<(X_{2},B_{2}\right)$ means that $\left(X_{1},B_{1})\leq (X_{2},B_{2}\right)$ and $\left(X_{1},B_{1})\neq (X_{2},B_{2}\right)$ hold at the same time. If $\left(X_{1},B_{1})<(X_{2},B_{2}\right)$ and there does not exist a concept (Y,C) such that $\left(X_{1},B_{1})<(Y,C)<(X_{2},B_{2}\right)$, then $\left(X_{1},B_{1}\right)$ is called a child-concept(immediate sub-concept) of $\left(X_{2},B_{2}\right)$ and $\left(X_{2},B_{2}\right)$ is called a parent-concept (immediate super-concept) of $\left(X_{1},B_{1}\right)$, and this is denoted by $\left(X_{1},B_{1})≺(X_{2},B_{2}\right)$.

For any two concept $\left(X_{1},B_{1}\right)$ and $\left(X_{2},B_{2}\right)$ of a formal context (U,A,I), it is easily proved that $\left(X_{1}\cap X_{2},{\left(B_{1}\cup B_{2}\right)}^{**}\right)$ and $\left({\left(X_{1}\cup X_{2}\right)}^{**},B_{1}\cap B_{2}\right)$ are also both concepts. Hence, if the meet and join are given by (Ganter and Wille [39]):

$$(X_{1},B_{1})\wedge (X_{2},B_{2})=(X_{1}\cap X_{2},{\left(B_{1}\cup B_{2}\right)}^{**}),$$

$$(X_{1},B_{1})\vee (X_{2},B_{2})=({\left(X_{1}\cup X_{2}\right)}^{**},B_{1}\cap B_{2}).$$

then the concept lattice L(U,A,I) is complete lattice.

**Definition** **3.** *(Atanassov [42]) Let* U *be a finite and non-empty set, called universe. An IF set* $\tilde{A}$ *of* U *has the following form*

$$\tilde{A}=\left\{\langle x,\mu_{\tilde{A}}(x),\gamma_{\tilde{A}}(x)\rangle |x\in U\right\},$$

*where $\mu_{\tilde{A}}:U\to [0,1]$ and $\gamma_{\tilde{A}}:U\to [0,1]$, $\mu_{\tilde{A}}(x)$ and $\gamma_{\tilde{A}}(x)$ are, respectively, called the membership degree and non-membership degree to A of the object x∈U. Furthermore, they satisfy $0\leq \mu_{\tilde{A}}(x)+\gamma_{\tilde{A}}(x)\leq 1$ for any x∈U. In generally, we use IF(U) to denote all IF sets in the universe U.*

**Definition** **4.** *(Atanassov [43]) Let*$\tilde{A},\tilde{B}\in IF(U)$. $\tilde{A}\subseteq \tilde{B}\Leftrightarrow$$\mu_{\tilde{A}}(x)\leq \mu_{\tilde{B}}(x)$*and*$\gamma_{\tilde{A}}(x)\geq \gamma_{\tilde{B}}(x)$*for any*x∈U*.*

If both $\tilde{A}\subseteq \tilde{B}$ and $\tilde{B}\subseteq \tilde{A}$, then we say $\tilde{A}$ is equal to $\tilde{B}$, denoted by $\tilde{A}=\tilde{B}$. The universe set and empty set are special IF set where $\tilde{U}=\{<x,1,0>|x\in U\}$ and $\tilde{\emptyset }=\{<x,0,1>|x\in U\}$.

Let denote intersection and union of $\tilde{A}$ and $\tilde{B}$ by $\tilde{A}\cap \tilde{B}$ and $\tilde{A}\cup \tilde{B}$, respectively. Moreover, we denote complement of $\tilde{A}$ by $\sim \tilde{A}$.

**Definition** **5.** *(Atanassov [43]) Let*$\tilde{A},\tilde{B}\in IF(U)$, then

$$\begin{array}{l} \tilde{A}\cap \tilde{B}=\left\{\langle x,\wedge \{\mu_{\tilde{A}}(x),\mu_{\tilde{B}}(x)\},\vee \{\gamma_{\tilde{A}}\left(x\right),\gamma_{\tilde{B}}\left(x\right)\}\rangle |x\in U\right\},\\ \tilde{A}\cup \tilde{B}=\left\{\langle x,\vee \{\mu_{\tilde{A}}(x),\mu_{\tilde{B}}(x)\},\wedge \{\gamma_{\tilde{A}}\left(x\right),\gamma_{\tilde{B}}\left(x\right)\}\rangle |x\in U\right\},\\ \sim \tilde{A}=\left\{\langle x,\gamma_{\tilde{A}}\left(x\right),\mu_{\tilde{A}}\left(x\right)\rangle |x\in U\right\} \end{array}$$

Many properties of these operators in IF set theory are similar with fuzzy set theory. Detailed description can be easily found in relative reference [54].

Next, we will review some basic results of the IF formal context and IF decision formal context.

**Definition** **6.** *(Jinzhong Pang [53]) A triple* $\left(U,A,\tilde{I}\right)$ *is called an IF formal context, if* $U=\{x_{1},x_{2},\cdots ,x_{n}\}$ *is an object set, where* $x_{i}(i\leq n)$ *is called an object,* $A=\{a_{1},a_{2},\cdots ,a_{m}\}$ *is an attribute set, where* $a_{j}(j\leq m)$ *is called an attribute, and* $\tilde{I}$ *is an IF set of* U×A *, where* $\tilde{I}=\left\{\langle \left(x,a\right),\mu_{\tilde{I}}\left(x,a\right),\gamma_{\tilde{I}}\left(x,a\right)\rangle |\left(x,a\right)\in U\times A\right\}$ *,* $\mu_{\tilde{I}}:U\times A\to \left[0,1\right]$ *and* $\gamma_{\tilde{I}}:U\times A\to \left[0,1\right]$ *.*

The complement of $\tilde{I}$ is denoted by $\sim \tilde{I}=\left\{\langle \left(x,a\right),\gamma_{\tilde{I}}\left(x,a\right),\mu_{\tilde{I}}\left(x,a\right)\rangle |\left(x,a\right)\in U\times A\right\}$.

We denote $\tilde{I}\left(x,a\right)=\langle \mu_{\tilde{I}}\left(x,a\right),\gamma_{\tilde{I}}\left(x,a\right)\rangle$, then the set of $\tilde{I}\left(x,a\right)(x\in U,a\in A)$ is denoted by $V=\{\tilde{I}\left(x,a\right)|x\in U,a\in A\}$.

Let $\tilde{I}\left(x,a\right)$, $\tilde{I}\left(y,a\right)$ ∈ V, then

$$\tilde{I}\left(x,a\right)\geq \tilde{I}\left(y,a\right)\Leftrightarrow \mu_{\tilde{I}}\left(x,a\right)\geq \mu_{\tilde{I}}\left(y,a\right)\wedge \gamma_{\tilde{I}}\left(x,a\right)\leq \gamma_{\tilde{I}}(y,a).$$

With respect to an IF formal context $\left(U,A,\tilde{I}\right)$, for X⊆U, B⊆A and $\tilde{A}$, $\tilde{B}$ ∈IF(U), where $\forall b,\tilde{B}(b),\;\tilde{A}(b)\in \{\tilde{I}(x,b)|\forall x\in U\}$, and $V_{b}\in \{\tilde{I}(x,b)|\forall x\in U\}$. 

A pair of operators is defined by

$$X^{*}=\tilde{A}=\{<a,\mu_{X^{*}}(a),\gamma_{X^{*}}(a)>|a\in A\}.$$

where $\tilde{A}(a)=\langle \underset{\forall x\in X}{\wedge }\;\mu_{\tilde{I}}(x,a),\underset{\forall x\in X}{\vee }\;\gamma_{\tilde{I}}(x,a)\rangle (a\in A)$. We rule $\emptyset^{*}=\tilde{A}=$ {〈a,1,0〉|a∈A}.

$${\tilde{B}}^{*}=\{x\in U|\tilde{I}(x,b)\geq \tilde{B}(b),\forall b\in B\},$$

where $\tilde{I}(x,b)\in V$. We denote $\tilde{B}(b)=<0,1>$, if b∉B.

Similarly, ∀x∈U, we use $x^{*}$ and $a^{*}$ instead of ${\left\{x\right\}}^{*}$ and ${\left\{a\right\}}^{*}$ respectively, and for any B⊆A denote $U^{B}=$ $\left\{\tilde{A}|\tilde{A}\left(b\right)=\tilde{I}\left(x,b\right),x\in U,b\in B\right\}$.

**Proposition** **2.** *(Jinzhong Pang [53]) Let* $\left(U,A,\tilde{I}\right)$ *be an IF formal context,* $X_{1},X_{2},X\subseteq U$ *,* $B_{1},B_{2},B\subseteq A$ *, then the above operators have the following properties.*

(1) $X_{1}\subseteq X_{2}\Rightarrow X_{2}^{*}\subseteq X_{1}^{*},\;{\tilde{B}}_{1}\subseteq {\tilde{B}}_{2}\Rightarrow {\tilde{B}}_{2}^{*}\subseteq {\tilde{B}}_{1}^{*}.$
(2) $X\subseteq X^{**},\;\tilde{B}\subseteq {\tilde{B}}^{**}.$
(3) $X^{*}=X^{***},\;{\tilde{B}}^{*}={\tilde{B}}^{***}.$
(4) $X\subseteq {\tilde{B}}^{*}\Leftrightarrow \tilde{B}\subseteq X^{*}.$
(5) ${\left(X_{1}\cup X_{2}\right)}^{*}=X_{1}^{*}\cap X_{2}^{*},\;{\left({\tilde{B}}_{1}\cup {\tilde{B}}_{2}\right)}^{*}={\tilde{B}}_{1}^{*}\cap {\tilde{B}}_{2}^{*}.$
(6) ${\left(X_{1}\cap X_{2}\right)}^{*}⊇X_{1}^{*}\cup X_{2}^{*},\;{\left({\tilde{B}}_{1}\cap {\tilde{B}}_{2}\right)}^{*}⊇{\tilde{B}}_{1}^{*}\cup {\tilde{B}}_{2}^{*}.$

**Definition** **7.** *(Jinzhong Pang [53]) Let*$\left(U,A,\tilde{I}\right)$*be an IF formal context. A pair*$\left(X,\tilde{B}\right)$*is called an IF formal concept (in brief a concept) if*$X^{*}=\tilde{B}$*and*$X={\tilde{B}}^{*}$forX⊆U*,*B⊆A. X*and*$\tilde{B}$*are called the extension and the intension of*$\left(X,\tilde{B}\right)$, *respectively.*

From the above, it is clear that both $\left(X^{**},X^{*}\right)$ and $\left({\tilde{B}}^{*},{\tilde{B}}^{**}\right)$ are concepts.

IF concept lattice $L(U,A,\tilde{I})$ is referred to all concepts of an IF formal context $\left(U,A,\tilde{I}\right)$, and they are ordered by

$$(X_{1},{\tilde{B}}_{1})\leq (X_{2},{\tilde{B}}_{2})\Leftrightarrow X_{1}\subseteq X_{2}\Leftrightarrow {\tilde{B}}_{1}⊇{\tilde{B}}_{2}.$$

where $\left(X_{1},{\tilde{B}}_{1}\right)$ and $\left(X_{2},{\tilde{B}}_{2}\right)$ are concepts. $\left(X_{1},{\tilde{B}}_{1}\right)$ is called a sub-concept of $\left(X_{2},{\tilde{B}}_{2}\right)$, and $\left(X_{2},{\tilde{B}}_{2}\right)$ is called a super-concept of $\left(X_{1},{\tilde{B}}_{1}\right)$.

**Proposition** **3.** (Jinzhong Pang [53]) If $\left(X_{1},{\tilde{B}}_{1}\right)$ and $\left(X_{2},{\tilde{B}}_{2}\right)$ are two concepts of an IF formal context $\left(U,A,\tilde{I}\right)$, then $\left(X_{1}\cap X_{2},{\left(B_{1}\cup B_{2}\right)}^{**}\right)$ and $\left({\left(X_{1}\cup X_{2}\right)}^{**},B_{1}\cap B_{2}\right)$ are also both concepts.

Hence, from the above, if the meet and join are given by:

$$(X_{1},{\tilde{B}}_{1})\wedge (X_{2},{\tilde{B}}_{2})=(X_{1}\cap X_{2},{\left({\tilde{B}}_{1}\cup {\tilde{B}}_{2}\right)}^{**}),$$

$$(X_{1},{\tilde{B}}_{1})\vee (X_{2},{\tilde{B}}_{2})=({\left(X_{1}\cup X_{2}\right)}^{**},B_{1}\cap {\tilde{B}}_{2}),$$

then the IF concept lattice $L(U,A,\tilde{I})$ is complete lattice. 

Let $\left(U,A,\tilde{I}\right)$ be an IF formal context and D⊆A. We denote ${\tilde{I}}_{D}=\tilde{I}\cap {\tilde{I}}^{\prime }$, where ${\tilde{I}}^{\prime }$ is an IF set of U×D, that is, ${\tilde{I}}_{D}=\left\{\langle \left(x,a),\mu_{{\tilde{I}}_{D}}(x,a),\gamma_{{\tilde{I}}_{D}}(x,a\right)\rangle |(x,a)\in U\times D\right\}$. Obviously, $\left(U,D,{\tilde{I}}_{D}\right)$ is also an IF formal context, we denote all concepts of a formal context $\left(U,D,{\tilde{I}}_{D}\right)$ by $L(U,D,{\tilde{I}}_{D})$ similarly. For $\forall (X,\tilde{B})\in L(U,D,{\tilde{I}}_{D})$, it satisfies that if a∈D, $X^{{*}_{D}}\left(a\right)=X^{*}\left(a\right)$ otherwise $X^{{*}_{D}}\left(a\right)=\langle 0,1\rangle$, and ${\tilde{B}}^{{*}_{D}}=$ $\left\{x\in U|\tilde{I}(x,b)\geq \tilde{B}(b),\forall b\in D\right\}$.

**Definition** **8.** *(Jinzhong Pang [53]) Let $L(U,A_{1},{\tilde{I}}_{1})$ and $L(U,A_{2},{\tilde{I}}_{2})$ be two IF concept lattices. If for any $\left(X,\tilde{B})\in L(U,A_{2},{\tilde{I}}_{2}\right)$, there exists $\left(X^{\prime },\tilde{B^{\prime }})\in L(U,A_{1},{\tilde{I}}_{1}\right)$, such that $X=X^{\prime }$, then we say that $L(U,A_{2},{\tilde{I}}_{2})$ is coarser than $L(U,A_{1},{\tilde{I}}_{1})$, denoted by $L(U,A_{1},{\tilde{I}}_{1})\leq L(U,A_{2},{\tilde{I}}_{2}).$*

If $L(U,A_{1},{\tilde{I}}_{1})\leq L(U,A_{2},{\tilde{I}}_{2})$ and $L(U,A_{2},{\tilde{I}}_{2})\leq L(U,A_{1},{\tilde{I}}_{1})$, then we say that $L(U,A_{1},{\tilde{I}}_{1})$ and $L(U,A_{2},{\tilde{I}}_{2})$ are isomorphic with each other, and are denoted by $L(U,A_{1},{\tilde{I}}_{1})≅L(U,A_{2},{\tilde{I}}_{2})$. We denote the family of all IF concept lattices by $L=\{L(U,A,\tilde{I})|(U,A,\tilde{I})\;\text{is an IF formal context}\}$.

**Definition** **9.** *(Jinzhong Pang [53]) A quintuple* $\left(U,A,\tilde{I},T,\tilde{J}\right)$ *is called an IF decision formal context, if* $\left(U,A,\tilde{I}\right)$ *and* $\left(U,T,\tilde{J}\right)$ *are IF formal context, where* A∩T=∅ *,* $\tilde{I}\subseteq U\times A$ *,* $\tilde{J}\subseteq U\times T$ *, and* A *and* T *are called the conditional attribute set and decision attribute set, respectively.*

We say that $L(U,A,\tilde{I})$ is the IF concept lattice of $\left(U,A,\tilde{I}\right)$ and $L(U,T,\tilde{J})$ is the IF concept lattice of $\left(U,T,\tilde{J}\right)$.

**Definition** **10.** *(Jinzhong Pang [53]) Let* $\left(U,A,\tilde{I},T,\tilde{J}\right)$ *be an IF decision formal context. The set of all extensions of* $L(U,A,\tilde{I})$ *and* $L(U,T,\tilde{J})$ *are defined to be* $L_{U}(U,A,\tilde{I})=\left\{X|(X,\tilde{B})\in L(U,A,\tilde{I})\right\}$ *and* $L_{U}(U,T,\tilde{J})=\left\{X|(X,\tilde{F})\in L(U,T,\tilde{J})\right\}$ *, respectively.*

**Proposition** **4.** *(Jinzhong Pang [53]) Let* $\left(U,A,\tilde{I},T,\tilde{J}\right)$ *be an IF decision formal context. If* X⊆U *,* D⊆A *and* $\tilde{B}\in IF(U)$ *, then* $X^{{*}_{D}}\subseteq X^{*}$ *and* ${\tilde{B}}^{*}\subseteq {\tilde{B}}^{{*}_{D}}$ *hold.*

**Definition** **11.** *(Jinzhong Pang [53]) Let* $\left(U,A,\tilde{I},T,\tilde{J}\right)$ *be an IF decision formal context, if* $L(U,A,\tilde{I})\leq L(U,T,\tilde{J})$ *, then* $\left(U,A,\tilde{I},T,\tilde{J}\right)$ *is called consistent.*

## 3. Attribute Reduction in IF Generalized Consistent Decision Formal Context

In this section, the definition of IF generalized consistent decision formal context is proposed and some important properties are discussed. Similar to the case in the classical decision formal context and the IF consistent decision formal context, attribute reduction in the IF generalized consistent decision formal context is still the key issue that needs to be investigated.

The paper [53] analyzed the knowledge reduction in the IF consistent decision formal context, which is based on the equivalence relation between conditional attribute concepts and decision attribute concepts. However, it only needs to satisfy the implication relation when dealing with data in real life. Accordingly, generalizing the notion of coarser is required, which can ascertain the implication relation between the conditional attribute concepts and the decision attribute concepts. The attribute reduction that is based on the generalized coarser will be investigated in this section.

### 3.1. The Basic Definitions and Propositions

**Definition** **12.** *Let* $L(U,A_{1},{\tilde{I}}_{1})$ *and* $L(U,A_{2},{\tilde{I}}_{2})$ *be two IF concept lattices,* $\left(X,\tilde{B})\in L(U,A_{2},{\tilde{I}}_{2}\right)$ *,* $\left(X^{\prime },\tilde{B^{\prime }})\in L(U,A_{1},{\tilde{I}}_{1}\right)$ *. If* $X^{\prime }\subseteq X$ *, then we say that* $\left(X^{\prime },\tilde{B^{\prime }}\right)$ *implies* $\left(X,\tilde{B}\right)$ *, denoted by* $\left(X^{\prime },\tilde{B^{\prime }})\to (X,\tilde{B}\right)$ *.*

**Definition** **13.** *Let* $L(U,A_{1},{\tilde{I}}_{1})$ *and* $L(U,A_{2},{\tilde{I}}_{2})$ *be two IF concept lattices. If there exists an injection* f *:* $L(U,A_{2},{\tilde{I}}_{2})\to L(U,A_{1},{\tilde{I}}_{1})$ *, such that*

(1) f((U,∅))=(U,∅) *,* $f((\emptyset ,{\tilde{A}}_{2}))=(\emptyset ,{\tilde{A}}_{1})$ *,*
(2) $\forall (X,\tilde{B})\in L(U,A_{2},{\tilde{I}}_{2})$ *,* $f((X,\tilde{B}))\to (X,\tilde{B})$ *,*

*then f is called an implication mapping from $L(U,A_{2},{\tilde{I}}_{2})$ to $L(U,A_{1},{\tilde{I}}_{1})$.*

The set of all concepts’ extensions in the range of f are defined to be $L_{U}^{f}(U,A_{1},{\tilde{I}}_{1})=$ $\left\{X^{\prime }|(X^{\prime },\tilde{B^{\prime }})=f((X,\tilde{B})),(X,\tilde{B})\in L(U,A_{2},{\tilde{I}}_{2})\right\}$ and it is obviously that $L_{U}^{f}(U,A_{1},{\tilde{I}}_{1})\subseteq L_{U}(U,A_{1},{\tilde{I}}_{1})$.

It is obvious that $U,\emptyset \in L_{U}^{f}(U,A_{1},{\tilde{I}}_{1})$, $\left|L_{U}^{f}(U,A_{1},{\tilde{I}}_{1})|=|L_{U}(U,A_{2},{\tilde{I}}_{2})\right|$ and every element of $L_{U}^{f}(U,A_{1},{\tilde{I}}_{1})$ is included in the corresponding element of $L_{U}(U,A_{2},{\tilde{I}}_{2})$ under the implication mapping, denoted by $L_{U}^{f}(U,A_{1},{\tilde{I}}_{1})⋐L_{U}(U,A_{2},{\tilde{I}}_{2})$

It is obvious that the following proposition holds.

**Proposition** **5.** *Let* $L(U,A_{1},{\tilde{I}}_{1})$ *and* $L(U,A_{2},{\tilde{I}}_{2})$ *be two IF concept lattices. Subsequently, there exists an implication mapping* f *:* $L(U,A_{2},{\tilde{I}}_{2})\to L(U,A_{1},{\tilde{I}}_{1})$ $\Leftrightarrow \exists L^{\prime }\subseteq L_{U}(U,A_{1},{\tilde{I}}_{1})$ *,* $L^{\prime }⋐L_{U}(U,A_{2},{\tilde{I}}_{2})$ *.*

**Definition** **14.** *Let*$L(U,A_{1},{\tilde{I}}_{1})$*and*$L(U,A_{2},{\tilde{I}}_{2})$*be two IF concept lattices. If there exists an implication mapping*f*:*$L(U,A_{2},{\tilde{I}}_{2})\to L(U,A_{1},{\tilde{I}}_{1})$*, we say that*$L(U,A_{1},{\tilde{I}}_{1})$*is generalized coarser than*$L(U,A_{2},{\tilde{I}}_{2})$*, denoted by*$L(U,A_{1},{\tilde{I}}_{1})≼L(U,A_{2},{\tilde{I}}_{2})$.

Obviously, the relation of coarser in Definition 8 is the special case and the following proposition holds.

**Proposition** **6.** *Let* $\left(U,A,\tilde{I}\right)$ *be an IF formal context. If* D⊆A *,* D≠∅ *, then there must exist the following relation* $L(U,A,\tilde{I})≼L(U,D,{\tilde{I}}_{D})$ *.*

**Proposition** **7.** *Let* $L(U,A_{1},{\tilde{I}}_{1})$ *and* $L(U,A_{2},{\tilde{I}}_{2})$ *be two IF concept lattices.* $L(U,A_{1},{\tilde{I}}_{1})≼L(U,A_{2},{\tilde{I}}_{2})$ $\Leftrightarrow \exists L^{\prime }\subseteq L_{U}(U,A_{1},{\tilde{I}}_{1})$ *,* $L^{\prime }⋐L_{U}(U,A_{2},{\tilde{I}}_{2})$

**Proposition** **8.** *Let* $L(U,A_{1},{\tilde{I}}_{1})$ *and* $L(U,A_{2},{\tilde{I}}_{2})$ *be two IF concept lattices. If* $L(U,A_{1},{\tilde{I}}_{1})≼$ $L(U,A_{2},{\tilde{I}}_{2})$ *and* $L(U,A_{2},{\tilde{I}}_{2})≼L(U,A_{1},{\tilde{I}}_{1})$ *hold, then* $L_{U}(U,A_{1},{\tilde{I}}_{1})=L_{U}(U,A_{2},{\tilde{I}}_{2})$ *.*

**Proof.**  Suppose that $L(U,A_{1},{\tilde{I}}_{1})≼L(U,A_{2},{\tilde{I}}_{2})$ and $L(U,A_{2},{\tilde{I}}_{2})≼L(U,A_{1},{\tilde{I}}_{1})$, and then there exist two implication mappings f:$L(U,A_{2},{\tilde{I}}_{2})\to L(U,A_{1},{\tilde{I}}_{1})$, g:$L(U,A_{1},{\tilde{I}}_{1})\to L(U,A_{2},{\tilde{I}}_{2})$. Since f,g are two injections, $L(U,A_{1},{\tilde{I}}_{1})$ and $L(U,A_{2},{\tilde{I}}_{2})$ are two finite sets, $\left|L(U,A_{1},{\tilde{I}}_{1})|=|L(U,A_{2},{\tilde{I}}_{2})\right|$. It follows that f,g are surjections, and so they are bijections. Next, we suffice $L_{U}(U,A_{1},{\tilde{I}}_{1})=L_{U}(U,A_{2},{\tilde{I}}_{2})$. □

Firstly, we have $f((\emptyset ,{\tilde{A}}_{2}))=(\emptyset ,{\tilde{A}}_{1})$, $g((\emptyset ,{\tilde{A}}_{1}))=(\emptyset ,{\tilde{A}}_{2})$ according to the Definition 13.

Secondly, assume that $\left(X,\tilde{B})\in L(U,A_{2},{\tilde{I}}_{2}\right)$, $\left(X^{\prime },\tilde{B^{\prime }})\in L(U,A_{1},{\tilde{I}}_{1}\right)$, $\left(X,\tilde{B}\right)$ is the father concept of $\left(\emptyset ,{\tilde{A}}_{2}\right)$, $\left(X^{\prime },\tilde{B^{\prime }})=f((X,\tilde{B})\right)$, so we can obtain $\left(X^{\prime },\tilde{B^{\prime }})\neq (\emptyset ,{\tilde{A}}_{1}\right)$, $X^{\prime }\subseteq X$. Suppose that $\left(Y,\tilde{C})\in L(U,A_{2},{\tilde{I}}_{2}\right)$, $\left(Y,\tilde{C})=g((X^{\prime },\tilde{B^{\prime }})\right)$, it follows that $\left(Y,\tilde{C})\neq (\emptyset ,{\tilde{A}}_{2}\right)$, $Y\subseteq X^{\prime }\subseteq X$. Since $\left(X,\tilde{B}\right)$ is the father concept of $\left(\emptyset ,{\tilde{A}}_{2}\right)$, $\left(Y,\tilde{C})=(X,\tilde{B}\right)$ holds. Thus, $X^{\prime }=X$. Likewise, $\left(X^{\prime },\tilde{B^{\prime }}\right)$ is the father concept of $\left(\emptyset ,{\tilde{A}}_{1}\right)$. If studying the father concept of $\left(X,\tilde{B}\right)$, we can obtain the similar conclusion. By analogy, we can conclude that $L_{U}(U,A_{1},{\tilde{I}}_{1})=L_{U}(U,A_{2},{\tilde{I}}_{2})$.

**Corollary** **1.** *Let $L=\{L(U,A,\tilde{I})|(U,A,\tilde{I})\;\text{is an IF formal context}\}$, then (L,≼) is a partial ordered set.*

**Definition** **15.** *Let*$\left(U,A,\tilde{I},T,\tilde{J}\right)$*be an IF decision formal context. Subsequently,*$\left(U,A,\tilde{I},T,\tilde{J}\right)$*is called generalized consistent, if*$L(U,A,\tilde{I})≼L(U,T,\tilde{J})$.

It is obvious that IF decision consistent formal context proposed in paper [53] is generalized consistently.

**Proposition** **9.** *Let* $\left(U,A,\tilde{I},T,\tilde{J}\right)$ *be an IF decision formal context, then the following three propositions are equivalent:*

(1) *$\left(U,A,\tilde{I},T,\tilde{J}\right)$ generalized consistent.*
(2) *There exists an implication mapping f: $L(U,T,\tilde{J})\to L(U,A,\tilde{I})$.*
(3) *$\exists L^{\prime }\subseteq L_{U}(U,A,\tilde{I}),L^{\prime }⋐L_{U}(U,T,\tilde{J})$.*

**Proof.**  It can be easily obtained from above discussions. □

### 3.2. Attribute Reductions in View of the Implication Mapping

**Definition** **16.** *Let* $\left(U,A,\tilde{I},T,\tilde{J}\right)$ *be an IF generalized consistent decision formal context,* f *:* $L(U,T,\tilde{J})\to L(U,A,\tilde{I})$ *be an implication mapping,* D⊆A *. We say that* D *is a consistent set of* $\left(U,A,\tilde{I},T,\tilde{J}\right)$ *based on* f *, if* $L_{U}^{f}(U,A,\tilde{I})\subseteq L_{U}(U,D,{\tilde{I}}_{D})$ *. Furthermore, if for any* d∈D *,* $L_{U}^{f}(U,A,\tilde{I})\subseteq$ $L_{U}(U,D-\{d\},{\tilde{I}}_{D-\{d\}})$ *does not hold, and then* D *is called an attribute reduction of* $\left(U,A,\tilde{I},T,\tilde{J}\right)$ *based on* f *. The intersection set of all reductions is called core of* $\left(U,A,\tilde{I},T,\tilde{J}\right)$ *based on* f *.*

**Definition** **17.** *Let* $\left(U,A,\tilde{I},T,\tilde{J}\right)$ *be an IF generalized consistent decision formal context,* f *:* $L(U,T,\tilde{J})\to L(U,A,\tilde{I})$ *is an implication mapping and suppose that* τ *is an index set and all of the reductions denoted by* $\left\{D^{i}|D^{i}is\;a\;reduction,\;i\in \tau \right\}$ *. Afterwards, conditional attributes can be classified four sorts based on* f *as follows:*

(1) *Absolutely necessary attribute (core attribute)* $b:b\in \underset{i\in \tau }{\cap }D^{i}$ *,*
(2) *Relatively necessary attribute* $c:c\in \underset{i\in \tau }{\cup }D^{i}-\underset{i\in \tau }{\cap }D^{i}$ *,*
(3) *Absolutely unnecessary attribute* $d:d\in A-\underset{i\in \tau }{\cup }D^{i}$ *,*
(4) *Unnecessary attribute* $e:e\in A-\underset{i\in \tau }{\cap }D^{i}$ *.*

**Proposition** **10.** *Let* $\left(U,A,\tilde{I},T,\tilde{J}\right)$ *be an IF generalized consistent decision formal context* f,g *are two implication mappings. The reduction based on* f *is the same with that based on* g *, if* $L_{U}^{f}(U,A,\tilde{I})=L_{U}^{g}(U,A,\tilde{I})$ *.*

**Proof.**  It is easy to be verified.

Obviously, we can obtain the following propositions by the above definitions. □

**Proposition** **11.** *Let*$\left(U,A,\tilde{I},T,\tilde{J}\right)$*be an IF generalized consistent decision formal context and*f*:*$L(U,T,\tilde{J})\to L(U,A,\tilde{I})$*is an implication mapping. If*D⊆A*and*D≠∅*, then*D*is a consistent set based on*f$\Rightarrow L(U,A,\tilde{I})≼L(U,D,{\tilde{I}}_{D})≼L(U,T,\tilde{J})$.

**Proof.**  According to Proposition 3.15. and Definition 16, the conclusion can be easily obtained. □

**Proposition** **12.** *Let*$\left(U,A,\tilde{I},T,\tilde{J}\right)$*be an IF generalized consistent decision formal context and*f*:*$L(U,T,\tilde{J})\to L(U,A,\tilde{I})$*is an implication mapping, then there must exist a reduction of*$\left(U,A,\tilde{I},T,\tilde{J}\right)$*based on*f.

**Proof.**  If for any a∈A, $L_{U}^{f}(U,A,\tilde{I})\subseteq L_{U}(U,A-\{a\},{\tilde{I}}_{A-\{a\}})$ does not hold, and then A is its reduction. If there exists an attribute a∈A such that $L_{U}^{f}(U,A,\tilde{I})\subseteq L_{U}(U,A-\{a\},{\tilde{I}}_{A-\{a\}})$, then we study $B_{1}=A-\{a\}$. Further, if $\forall b_{1}\in B_{1}$ such that $L_{U}^{f}(U,A,\tilde{I})⊄L_{U}(U,B_{1}-\{b_{1}\},{\tilde{I}}_{B_{1}-\{b_{1}\}})$, and then $B_{1}$ is a reduction. Otherwise, we study $B_{1}-\{b_{1}\}$. Repeating the above process, we can find one reduction at least because A is a finite set. Thus, the reduction of $\left(U,A,\tilde{I},T,\tilde{J}\right)$ must exist.

In general, the reduction of $\left(U,A,\tilde{I},T,\tilde{J}\right)$ is not unique. □

**Example** **1.** *An example of an IF decision formal context*$\left(U,A,\tilde{I},T,\tilde{J}\right)$*is depicted in Table 1. In this context,*$U=\{x_{1},x_{2},x_{3},x_{4}\}$*,*A={a,b,c,d,e}*and*T={f,g,h}.

We can find all concepts of the $\left(U,A,\tilde{I}\right)$ by the definition, which are $\left(1,{\tilde{A}}_{1}\right)$, $\left(2,{\tilde{A}}_{2}\right)$, $\left(4,{\tilde{A}}_{3}\right)$, $\left(12,{\tilde{A}}_{4}\right)$, $\left(13,{\tilde{A}}_{5}\right)$, $\left(14,{\tilde{A}}_{6}\right)$, $\left(24,{\tilde{A}}_{7}\right)$, $\left(123,{\tilde{A}}_{8}\right)$, $\left(124,{\tilde{A}}_{9}\right)$, $\left(U,{\tilde{A}}_{10}\right)$, and $\left(\emptyset ,\tilde{A}\right)$ respectively, and we denote objects set $\left\{x_{i},x_{j}\right\}$ by ij(i,j=1,2,3,4), which is same to others, where

$$\begin{array}{l} {\tilde{A}}_{1}=\{\left(a,0.9,0.0\right),\left(b,0.7,0.2\right),\left(c,0.2,0.5\right),\left(d,0.9,0.1\right),\left(e,0.8,0.1\right)\},\\ {\tilde{A}}_{2}=\{\left(a,0.8,0.1\right),\left(b,0.8,0.2\right),\left(c,0.8,0.1\right),\left(d,0.3,0.5\right),\left(e,0.2,0.7\right)\},\\ {\tilde{A}}_{3}=\{\left(a,0.7,0.2\right),\left(b,0.8,0.1\right),\left(c,0.7,0.1\right),\left(d,0.2,0.6\right),\left(e,0.1,0.6\right)\},\\ {\tilde{A}}_{4}=\{\left(a,0.8,0.1\right),\left(b,0.7,0.2\right),\left(c,0.2,0.5\right),\left(d,0.3,0.5\right),\left(e,0.2,0.7\right)\},\\ {\tilde{A}}_{5}=\{\left(a,0.1,0.8\right),\left(b,0.7,0.3\right),\left(c,0.1,0.9\right),\left(d,0.8,0.2\right),\left(e,0.2,0.7\right)\},\\ {\tilde{A}}_{6}=\{\left(a,0.7,0.2\right),\left(b,0.7,0.2\right),\left(c,0.2,0.5\right),\left(d,0.2,0.6\right),\left(e,0.1,0.6\right)\},\\ {\tilde{A}}_{7}=\{\left(a,0.7,0.2\right),\left(b,0.8,0.2\right),\left(c,0.7,0.1\right),\left(d,0.2,0.6\right),\left(e,0.1,0.7\right)\},\\ {\tilde{A}}_{8}=\{\left(a,0.1,0.8\right),\left(b,0.7,0.3\right),\left(c,0.1,0.9\right),\left(d,0.3,0.5\right),\left(e,0.2,0.7\right)\},\\ {\tilde{A}}_{9}=\{\left(a,0.7,0.2\right),\left(b,0.7,0.2\right),\left(c,0.2,0.5\right),\left(d,0.2,0.6\right),\left(e,0.1,0.7\right)\},\\ {\tilde{A}}_{10}=\{\left(a,0.1,0.8\right),\left(b,0.7,0.3\right),\left(c,0.1,0.9\right),\left(d,0.2,0.6\right),\left(e,0.1,0.7\right)\},\\ \tilde{A}=\{\left(a,1,0\right),\left(b,1,0\right),\left(c,1,0\right),(d,1,0),(e,1,0)\}. \end{array}$$

**Figure 1:**
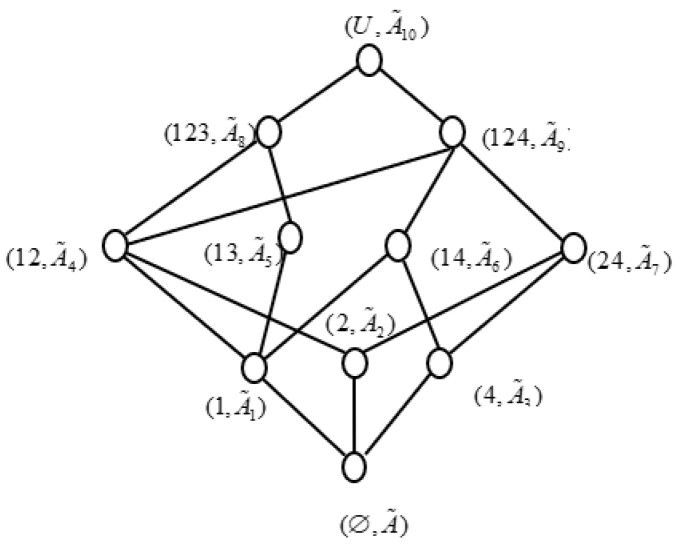
Concept lattice of $\left(U,A,\tilde{I}\right)$.

Furthermore, we can obtain IF concept lattice of $\left(U,A,\tilde{I}\right)$, as shown as Figure 1.

Similarly, all the concepts of $\left(U,T,\tilde{J}\right)$ can be obtained, which are $\left(2,{\tilde{T}}_{1}\right)$, $\left(4,{\tilde{T}}_{2}\right)$, $\left(24,{\tilde{T}}_{3}\right)$, $\left(124,{\tilde{T}}_{4}\right)$, $\left(134,{\tilde{T}}_{5}\right)$, $\left(U,{\tilde{T}}_{6}\right)$, and $\left(\emptyset ,{\tilde{T}}_{7}\right)$, respectively, where

$$\begin{array}{l} {\tilde{T}}_{1}=\{\left(f,0.8,0.2\right),\left(g,0.3,0.7\right),\left(h,0.5,0.2\right)\},\;{\tilde{T}}_{2}=\{\left(f,0.8,0.1\right),\left(g,0.7,0.1\right),\left(h,0.7,0.2\right)\},\\ {\tilde{T}}_{3}=\{\left(f,0.8,0.1\right),\left(g,0.4,0.5\right),\left(h,0.6,0.2\right)\},\;{\tilde{T}}_{4}=\{\left(f,0.2,0.8\right),\left(g,0.1,0.8\right),\left(h,0.6,0.3\right)\},\\ {\tilde{T}}_{5}=\{\left(f,0.8,0.2\right),\left(g,0.3,0.7\right),\left(h,0.5,0.2\right)\},\;{\tilde{T}}_{6}=\{\left(f,0.2,0.8\right),\left(g,0.1,0.8\right),\left(h,0.5,0.3\right)\},\\ {\tilde{T}}_{7}=\{\left(f,1,0\right),\left(g,1,0\right),\left(h,1,0\right)\}. \end{array}$$

**Figure 2:**
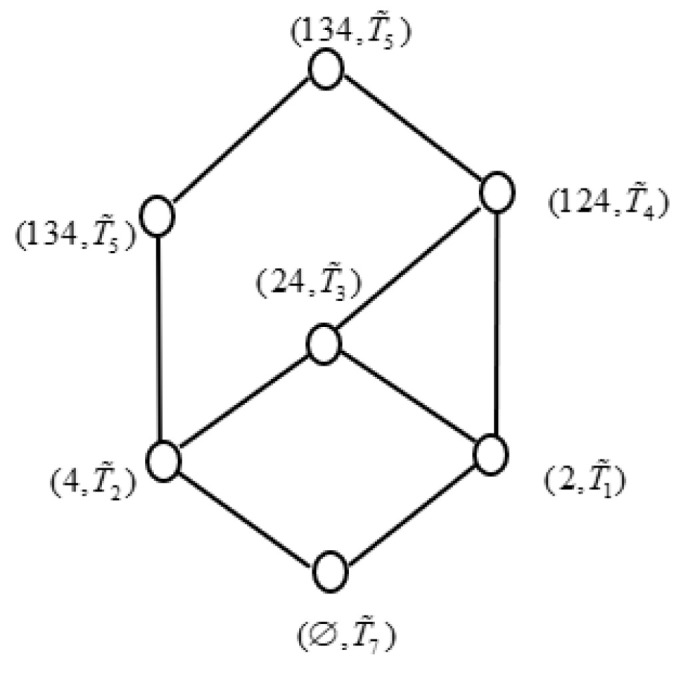
Concept lattice of $\left(U,T,\tilde{J}\right)$.

Similarly, the IF concept lattice of $\left(U,T,\tilde{J}\right)$ can be obtained in the following Figure 2.

It can be easily testified that $\left(U,A,\tilde{I},T,\tilde{J}\right)$ is generalized consistently in Table 1, and we take an implication mapping f: $L(U,T,\tilde{J})\to L(U,A,\tilde{I})$ i.e., $\{((\emptyset ,{\tilde{T}}_{7}),(\emptyset ,\tilde{A})),((2,{\tilde{T}}_{1}),(2,{\tilde{A}}_{2})),((4,{\tilde{T}}_{2}),(4,{\tilde{A}}_{3})),$ $\left((24,{\tilde{T}}_{3}),(24,{\tilde{A}}_{7})),((124,{\tilde{T}}_{4}),(1,{\tilde{A}}_{1})),((134,{\tilde{T}}_{5}),(13,{\tilde{A}}_{5})),((U,{\tilde{T}}_{6}),(U,{\tilde{A}}_{10}))\right\}$. If we take out {a,c,e} from the attributes set A, then we can obtain a new IF formal context $\left(U,D^{1},{\tilde{I}}_{D^{1}}\right)$, where $D^{1}=A-\{a,c,e\}$. We can get all concepts of $\left(U,D^{1},{\tilde{I}}_{D^{1}}\right)$, which are $\left(1,{\tilde{D}}_{1}^{1}\right)$, $\left(2,{\tilde{D}}_{2}^{1}\right)$, $\left(4,{\tilde{D}}_{3}^{1}\right)$, $\left(12,{\tilde{D}}_{4}^{1}\right)$, $\left(13,{\tilde{D}}_{5}^{1}\right)$, $\left(24,{\tilde{D}}_{6}^{1}\right)$, $\left(123,{\tilde{D}}_{7}^{1}\right)$, $\left(124,{\tilde{D}}_{8}^{1}\right)$, $\left(U,{\tilde{D}}_{9}^{1}\right)$, and $\left(\emptyset ,{\tilde{D}}^{1}\right)$, respectively, where

$$\begin{array}{l} {\tilde{D}}_{1}^{1}=\{\left(b,0.7,0.2\right),\left(d,0.9,0.1\right)\},\;{\tilde{D}}_{2}^{1}=\{\left(b,0.8,0.2\right),\left(d,0.3,0.5\right)\},\\ {\tilde{D}}_{3}^{1}=\{\left(b,0.8,0.1\right),\left(d,0.2,0.6\right)\},\;{\tilde{D}}_{4}^{1}=\{\left(b,0.7,0.2\right),\left(d,0.3,0.5\right)\},\\ {\tilde{D}}_{5}^{1}=\{\left(b,0.7,0.3\right),\left(d,0.8,0.2\right)\},\;{\tilde{D}}_{6}^{1}=\{\left(b,0.8,0.2\right),\left(d,0.2,0.6\right)\},\\ {\tilde{D}}_{7}^{1}=\{\left(b,0.7,0.3\right),\left(d,0.3,0.5\right)\},\;{\tilde{D}}_{8}^{1}=\{\left(b,0.7,0.2\right),\left(d,0.2,0.6\right)\},\\ {\tilde{D}}_{9}^{1}=\{\left(b,0.7,0.3\right),\left(d,0.2,0.6\right)\},\;{\tilde{D}}^{1}=\{\left(b,1,0\right),\left(d,1,0\right)\}. \end{array}$$

**Figure 3:**
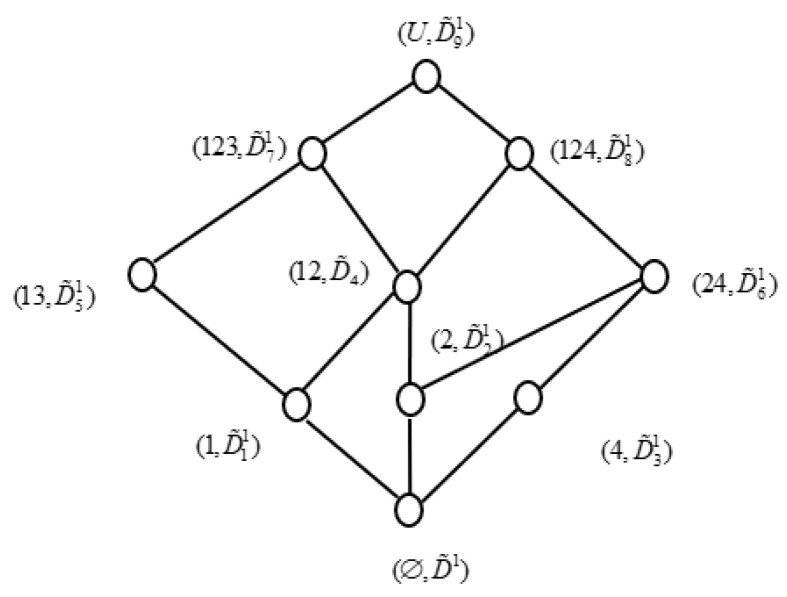
Concept lattice of $\left(U,D^{1},{\tilde{I}}_{D^{1}}\right)$.

In addition, concept lattice of I $\left(U,D^{1},{\tilde{I}}_{D^{1}}\right)$ can be obtained, as shown as Figure 3.

From Figure 1, Figure 2 and Figure 3, we can easily find that $L(U,D^{1},{\tilde{I}}_{D^{1}})≼L(U,T,\tilde{J})$. Accordingly, $D^{1}$ is a consistent set of $\left(U,A,\tilde{I},T,\tilde{J}\right)$. In fact, we can find $L(U,D^{1}-b,{\tilde{I}}_{D^{1}-b})≼L(U,T,\tilde{J})$, $L(U,D^{1}-d,$ ${\tilde{I}}_{D^{1}-d})$ $≼L(U,T,\tilde{J})$, $L(U,D^{1}-e,{\tilde{I}}_{D^{1}-e})≼L(U,T,\tilde{J})$ by calculating. Hence, $D^{1}$ is a reduction of $\left(U,A,\tilde{I},T,\tilde{J}\right)$.

Similarly, if we take out {a,b} from the attributes set A, then we can obtain a new IF formal context $\left(U,D^{2},{\tilde{I}}_{D^{2}}\right)$, where $D^{2}=A-\{a,b\}$ We can get all concepts of $\left(U,D^{2},{\tilde{I}}_{D^{2}}\right)$, they are $\left(1,{\tilde{D}}_{1}^{2}\right)$, $\left(2,{\tilde{D}}_{2}^{2}\right)$, $\left(4,{\tilde{D}}_{3}^{2}\right)$, $\left(12,{\tilde{D}}_{4}^{2}\right)$, $\left(13,{\tilde{D}}_{5}^{2}\right)$, $\left(14,{\tilde{D}}_{6}^{2}\right)$, $(24,{\tilde{D}}_{7}^{2}),$ $\left(123,{\tilde{D}}_{8}^{2}\right)$, $\left(124,{\tilde{D}}_{9}^{2}\right)$, $\left(U,{\tilde{D}}_{10}^{2}\right)$, and $\left(\emptyset ,{\tilde{D}}^{2}\right)$, respectively, where

$$\begin{array}{c} {\tilde{D}}_{1}^{2}=\{\left(c,0.2,0.5\right),\left(d,0.9,0.1\right),\left(e,0.8,0.1\right)\},\;{\tilde{D}}_{2}^{2}=\{\left(c,0.8,0.1\right),\left(d,0.3,0.5\right),\left(e,0.2,0.7\right)\},\\ {\tilde{D}}_{3}^{2}=\{\left(c,0.7,0.1\right),\left(d,0.2,0.6\right),\left(e,0.1,0.6\right)\},\;{\tilde{D}}_{4}^{2}=\{\left(c,0.2,0.5\right),\left(d,0.3,0.5\right),\left(e,0.2,0.7\right)\},\\ {\tilde{D}}_{5}^{2}=\{\left(c,0.1,0.9\right),\left(d,0.8,0.2\right),\left(e,0.2,0.7\right)\},\;{\tilde{D}}_{6}^{2}=\{\left(c,0.2,0.5\right),\left(d,0.2,0.6\right),\left(e,0.1,0.6\right)\},\\ {\tilde{D}}_{7}^{2}=\{\left(c,0.7,0.1\right),\left(d,0.2,0.6\right),\left(e,0.1,0.7\right)\},\;{\tilde{D}}_{8}^{2}=\{\left(c,0.1,0.9\right),\left(d,0.3,0.5\right),\left(e,0.2,0.7\right)\},\\ {\tilde{D}}_{9}^{2}=\{\left(c,0.2,0.5\right),\left(d,0.2,0.6\right),\left(e,0.1,0.7\right)\},\;{\tilde{D}}_{10}^{2}=\{\left(c,0.1,0.9\right),\left(d,0.2,0.6\right),\left(e,0.1,0.7\right)\},\\ {\tilde{D}}^{2}=\{\left(b,1,0\right),\left(d,1,0\right),\left(e,1,0\right)\}. \end{array}$$

**Figure 4:**
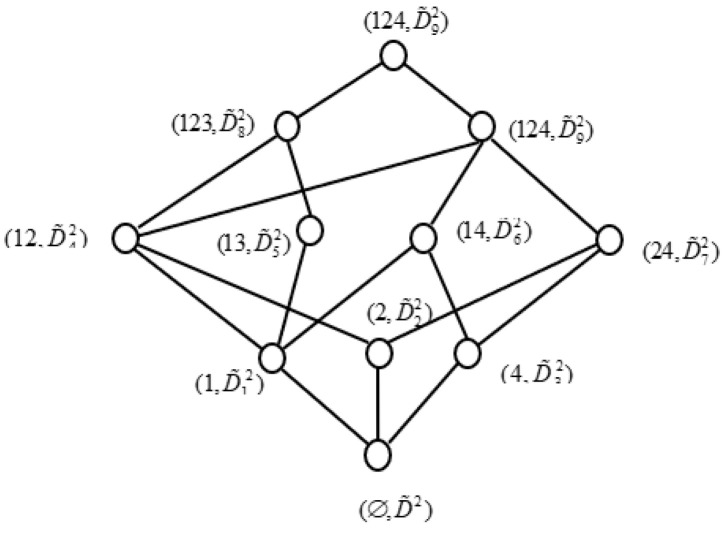
Concept lattice of $\left(U,D^{2},{\tilde{I}}_{D^{2}}\right)$.

In addition, we can obtain concept lattice of $\left(U,D^{2},{\tilde{I}}_{D^{2}}\right)$, as shown as Figure 4.

Furthermore, if taking the implication mapping f:$L(U,T,\tilde{J})\to L(U,A,\tilde{I})$ as $\{((\emptyset ,{\tilde{T}}_{7}),(\emptyset ,\tilde{A})),$ $\left((2,{\tilde{T}}_{1}),(2,{\tilde{A}}_{2})),((4,{\tilde{T}}_{2}),(4,{\tilde{A}}_{3})),((24,{\tilde{T}}_{3}),(24,{\tilde{A}}_{7})),((124,{\tilde{T}}_{4}),(1,{\tilde{A}}_{1})),((134,{\tilde{T}}_{5}),(14,{\tilde{A}}_{6})),((U,{\tilde{T}}_{6}),(U,{\tilde{A}}_{10}))\right\}$ and g:$L(U,T,\tilde{J})\to L(U,A,\tilde{I})$ as $\{((\emptyset ,{\tilde{T}}_{7}),(\emptyset ,\tilde{A}))((2,{\tilde{T}}_{1}),(2,{\tilde{A}}_{2})),((4,{\tilde{T}}_{2}),(4,{\tilde{A}}_{3})),((24,{\tilde{T}}_{3}),(24,{\tilde{A}}_{7})),$ $\left((124,{\tilde{T}}_{4}),(14,{\tilde{A}}_{6})),((134,{\tilde{T}}_{5}),(1,{\tilde{A}}_{1})),((U,{\tilde{T}}_{6}),(U,{\tilde{A}}_{10}))\right\}$. It can be easily verified that $L_{U}^{f}(U,A,\tilde{I})=$ $L_{U}^{g}(U,A,\tilde{I})$ i.e., the reduction based on f is same with that based on g.

**Corollary** **2.** *Let* $\left(U,A,\tilde{I},T,\tilde{J}\right)$ *be an IF generalized consistent decision formal context and* f *:* $L(U,T,\tilde{J})\to L(U,A,\tilde{I})$ *is an implication mapping. For* f *:*

*The core is the reduction* ⇔ *The reduction is only one.*

**Proof.**  ⇐Obviously.

⇒Assumed that the core is the reduction, and the reduction is not unique, that is, there are two reductions:$D^{i}\neq D^{j}$ at least. Hence, the core of the reductions $\cap D^{t}\subseteq D^{i}\cap D^{j}\subset D^{i}$. For is the reduction, the proper subset of it (where it is the core of the reductions) must not be the reduction. This clearly contradicts the known conditions. So, if the core is the reduction, the reduction is only one.

Obviously, the following corollaries can be obtained by the above definitions and propositions. □

**Corollary** **3.** *Let* $\left(U,A,\tilde{I},T,\tilde{J}\right)$ *be an IF generalized consistent decision formal context and* f *:* $L(U,T,\tilde{J})\to L(U,A,\tilde{I})$ *is an implication mapping. For* f *:*

*a*∈*A is a core attribute* ⇔ *A*-{*a*} *is not a consistent set.*

**Corollary** **4.** *Let*$\left(U,A,\tilde{I},T,\tilde{J}\right)$*be an IF generalized consistent decision formal context, and*f*:*$L(U,T,\tilde{J})\to L(U,A,\tilde{I})$*is an implication mapping. For*f:

*a*∈*A is an unnecessary attribute* ⇔ *A*-{*a*} *is a consistent set.*

Since the reduction D of an IF generalized consistent decision formal context based on f satisfies the following conditions: (1) D⊆A a consistent set. (2) ∀d∈D,D\{d} is not a consistent set. In order to get reductions, it is helpful to give the necessary and sufficient conditions of consistent sets in order to more easily obtain reductions.

**Proposition** **13.** *Let*$\left(U,A,\tilde{I},T,\tilde{J}\right)$*be an IF generalized consistent decision formal context,*D⊆A,D≠∅*and*f*:*$L(U,T,\tilde{J})\to L(U,A,\tilde{I})$*is an implication mapping. Subsequently, for*f:

$$D\;\text{is a consistent set of}\;(U,A,\tilde{I},T,\tilde{J})\Leftrightarrow X_{i}=X_{i}{}^{{*}_{D}{*}_{D}}\;\text{for any}\;X_{i}\in L_{U}^{f}(U,A,\tilde{I}).$$

**Proof.**  Assume that D is a consistent set, and then we have $L_{U}^{f}(U,A,\tilde{I})\subseteq L_{U}(U,D,{\tilde{I}}_{D})$, according to Definition 16. For any $X_{i}\in L_{U}^{f}(U,A,\tilde{I})$, it satisfies $X_{i}\in L_{U}(U,D,{\tilde{I}}_{D})$, which is to say that there exists $\tilde{B}\in U^{D}$, such that $\left(X_{i},\tilde{B})\in L(U,D,{\tilde{I}}_{D}\right)$. Hence, $X_{i}=X_{i}{}^{{*}_{D}{*}_{D}}$.

Conversely, it is obvious. □

**Corollary** **5.** *Let* $\left(U,A,\tilde{I},T,\tilde{J}\right)$ *be an IF generalized consistent decision formal context,* D⊆A,D≠∅ *, and* f *:* $L(U,T,\tilde{J})\to L(U,A,\tilde{I})$ *is an implication mapping. Subsequently, for* f *:*

$$D\;\text{is a consistent set of}\;(U,A,\tilde{I},T,\tilde{J})\Leftrightarrow \forall X_{i}\in L_{U}^{f}(U,A,\tilde{I}),\;\exists \tilde{B}\in U^{D}\;\text{such that}\;{\tilde{B}}^{{*}_{D}}=X_{i}.$$

In Definition 17, conditional attributes are classified four sorts, which are absolutely necessary attribute, relatively necessary attribute, absolutely unnecessary attribute, and unnecessary attribute based on the relation between conditional attributes and decision attributes. A different kind of attribute has a different effect in reduction. Next, some propositions of the attribute will be presented.

**Proposition** **14.** *Let* $\left(U,A,\tilde{I},T,\tilde{J}\right)$ *be an IF generalized consistent decision formal context, and* f *:* $L(U,T,\tilde{J})\to L(U,A,\tilde{I})$ *is an implication mapping. Subsequently, for* f *:* C *is a set of absolutely necessary attributes if* ∀a∈C *there exists* $X_{i}\in L_{U}^{f}(U,A,\tilde{I})$ *, such that* $X_{i}^{*}(a)>\tilde{I}(x_{j},a)$ *, and for any* b∉C $\tilde{I}(x_{j},b)\geq X_{i}^{*}(b)$ *.*

**Proof.**  We only need to prove that C={a}, because if C contains more one element, then we can treat C as one new attribute to deal with. Suppose that a is an unnecessary attribute, then D=A−{a} is a consistent set, i.e., $L_{U}^{f}(U,A,\tilde{I})\subseteq L_{U}(U,D,{\tilde{I}}_{D})$. For any $X_{i}\in L_{U}^{f}(U,A,\tilde{I})$, it satisfies $X_{i}\in L_{U}(U,D,{\tilde{I}}_{D})$. So, for any $X_{i}\in L_{U}^{f}(U,A,\tilde{I})$, $\exists {\tilde{B}}_{1}\in U^{A}$ and ${\tilde{B}}_{2}\in U^{D}$, $st(X_{i},{\tilde{B}}_{1})\in L(U,A,\tilde{I})$ and $\left(X_{i},{\tilde{B}}_{2})\in L(U,D,{\tilde{I}}_{D}\right)$.

However, ${\tilde{B}}_{1}(a)=X_{i}^{*}(a)>\tilde{I}(x_{j},a)$ i.e., $x_{j}\notin {\tilde{B}}_{1}^{{*}_{A}}=X_{i}$ and for any b≠a $\tilde{I}(x_{j},b)\geq X_{i}^{*}(b)$ i.e., $x_{j}\in {\tilde{B}}_{1}^{{*}_{D}}=X_{i}$ according to the above conditions, which comes a contradiction. Therefore, a is an absolutely necessary attribute. □

**Proposition** **15.** *Let*$\left(U,A,\tilde{I},T,\tilde{J}\right)$*be an IF generalized consistent decision formal context and*f*:*$L(U,T,\tilde{J})\to L(U,A,\tilde{I})$*be an implication mapping. Subsequently, for*f*:*a∈A*is an unnecessary attributes if the following conditions hold: For any*$X_{i}\in L_{U}^{f}(U,A,\tilde{I})$*and*$x_{j}\in U-X_{i}$*, if*$X_{i}^{*}(a)>\tilde{I}(x_{j},a)$*, then there exists*b≠a∈A*, such that*$X_{i}^{*}(b)>\tilde{I}(x_{j},b)$*. Moreover, if there exists*$x_{k}\in U-X_{i}$*such that*$\tilde{I}(x_{k},a)\geq X_{i}^{*}(a)$*, then*$\tilde{I}(x_{k},b)\geq X_{i}^{*}(b)$.

**Proof.**  Suppose that $a=a_{l}$, $D=A-\{a_{l}\}$. It suffices to prove that D is consistent set. By Corollary 5, it remains to prove that for any $X_{i}\in L_{U}^{f}(U,A,\tilde{I})$, there exists ${\tilde{B}}^{\prime }\in U^{D}$, such that $\tilde{B}\prime^{{*}_{D}}=X_{i}$. For any $X_{i}\in L_{U}^{f}(U,A,\tilde{I})$, there exists $\tilde{B}\in U^{A}$, such that $\left(X_{i},\tilde{B})\in L(U,A,\tilde{I}\right)$. So, suppose that $\tilde{B}=\left\{\tilde{I}\left(x_{s1},a_{1}\right),\tilde{I}\left(x_{s2},a_{2}\right),\cdots ,\tilde{I}\left(x_{sm},a_{m}\right)\right\}$, where $x_{st}\in U$ $a_{t}\in A$, 1≤s≤|U|, 1≤t≤m and $\tilde{B}(a_{l})=X_{i}^{*}(a_{l})=\tilde{I}\left(x_{sl},a_{l}\right)$.

If for any $x\in U-X_{i}$, $\tilde{I}\left(x,a_{l}\right)\geq \tilde{I}\left(x_{sl},a_{l}\right)$, then let ${\tilde{B}}^{\prime }=\{\tilde{I}\left(x_{s1},a_{1}\right),\tilde{I}\left(x_{s2},a_{2}\right),\cdots ,\tilde{I}\left(x_{sl-1},a_{l-1}\right),$ $\tilde{I}\left(x_{sl+1},a_{l+1}\right),\cdots ,\tilde{I}\left(x_{sm},a_{m}\right)\}$, so we can get $\tilde{B}\prime^{{*}_{D}}={\tilde{B}}^{*}$.

Otherwise, assume that there are $\{x_{t1},x_{t2},\cdots ,x_{t\alpha }\}\subseteq U-X_{i}$, such that $\tilde{I}\left(x_{sl},a_{l}\right)>\tilde{I}\left(x_{t\beta },a_{l}\right)$ (1≤β≤α), then there exist, according the condition, $a_{q1},a_{q2},\cdots ,a_{q\eta }\in A-\{a_{l}\}$ such that $\tilde{I}\left(x_{sl},a_{q\sigma }\right)>\tilde{I}\left(x_{t\beta },a_{q\sigma }\right)$ (1≤σ≤η). Moreover, if there exists $x_{k}$ such that $\tilde{I}\left(x_{k},a_{l}\right)\geq \tilde{I}\left(x_{sl},a_{l}\right)$, then $\tilde{I}\left(x_{k},a_{q\sigma }\right)\geq \tilde{I}\left(x_{sl},a_{q\sigma }\right)$. Let ${\tilde{B}}^{\prime }={\tilde{B}}_{1}\cup {\tilde{B}}_{2}$, where

$$\begin{array}{l} {\tilde{B}}_{1}=\{\tilde{I}\left(x_{s1},a_{1}\right),\tilde{I}\left(x_{s2},a_{2}\right),\cdots ,\tilde{I}\left(x_{sl-1},a_{l-1}\right),\tilde{I}\left(x_{sl+1},a_{l+1}\right),\cdots ,\tilde{I}\left(x_{sm},a_{m}\right)\},\\ {\tilde{B}}_{2}=\{\tilde{I}\left(x_{sl},a_{q1}\right),\tilde{I}\left(x_{sl},a_{q2}\right),\cdots ,\tilde{I}\left(x_{sl},a_{q\eta }\right)\}. \end{array}$$

then it follows that $\tilde{B}\prime^{{*}_{D}}={\tilde{B}}_{1}{}^{{*}_{D}}\cap {\tilde{B}}_{2}{}^{{*}_{D}}$. Hence, we know that, if $x\in {\tilde{B}}^{*}$, i.e., $\tilde{I}\left(x,a_{t}\right)\geq \tilde{I}\left(x_{st},a_{t}\right)$, then $\tilde{I}\left(x,a_{q\sigma }\right)\geq \tilde{I}\left(x_{sl},a_{q\sigma }\right)$. Subsequently, $x\in {\tilde{B}}_{1}{}^{*}$ and $x\in {\tilde{B}}_{2}{}^{*}$. It follows that $x\in \tilde{B}\prime^{{*}_{D}}$ and so ${\tilde{B}}^{*}\subseteq \tilde{B}\prime^{{*}_{D}}$. If $x\notin {\tilde{B}}^{*}$, i.e., there exists $a_{t_{0}}\in A$ such that $\tilde{I}\left(x_{st},a_{t_{0}}\right)>\tilde{I}\left(x,a_{t_{0}}\right)$. If $a_{t_{0}}=a_{l}$, i.e., $x\in \{x_{t1},x_{t2},\cdots ,x_{t\alpha }\}$, then $\tilde{I}\left(x_{sl},a_{q\sigma }\right)>\tilde{I}\left(x,a_{q\sigma }\right)$, i.e., $x\notin {\tilde{B}}_{2}{}^{{*}_{D}}$ and so $x\notin \tilde{B}\prime^{{*}_{D}}$. If $a_{t_{0}}\neq a_{l}$, i.e., then $\tilde{I}\left(x_{st_{0}},a_{t_{0}}\right)>\tilde{I}\left(x,a_{t_{0}}\right)$, i.e., $x\notin {\tilde{B}}_{1}{}^{{*}_{D}}$, and so $x\notin \tilde{B}\prime^{{*}_{D}}$. Hence, $\tilde{B}\prime^{{*}_{D}}={\tilde{B}}^{*}$.

Therefore, we conclude that for any $\tilde{F}\in U^{T}$, there exists ${\tilde{B}}^{\prime }\in U^{D}$, such that $\tilde{B}\prime^{{*}_{D}}={\tilde{F}}^{{*}_{T}}$. □

**Proposition** **16.** *Let* $\left(U,A,\tilde{I},T,\tilde{J}\right)$ *be an IF generalized consistent decision formal context and* f *:* $L(U,T,\tilde{J})\to L(U,A,\tilde{I})$ *is an implication mapping. Afterwards, for* f *:* a *is an unnecessary attribute if there exists* b≠a∈A *, such that: for any* $X_{i}\in L_{U}^{f}(U,A,\tilde{I})$ *and* $x_{j}\in U-X_{i}$ *, if* $X_{i}^{*}(a)>\tilde{I}(x_{j},a)$ *implies that* $X_{i}^{*}(b)>\tilde{I}(x_{j},b)$ *. Moreover, if* b *is an absolutely necessary attribute, then* a *is an absolutely unnecessary attribute.*

**Proof.**  Suppose that $a=a_{l}$, $b=a_{l-1}$, $D=A-\{a_{l}\}$. It suffices to prove that D is a consistent set. By Corollary 5. it remains to prove that, for any $X_{i}\in L_{U}^{f}(U,A,\tilde{I})$, there exists ${\tilde{B}}^{\prime }\in U^{D}$ such that $\tilde{B}\prime^{{*}_{D}}=X_{i}$. For any $X_{i}\in L_{U}^{f}(U,A,\tilde{I})$, there exists $\tilde{B}\in U^{A}$, such that $\left(X_{i},\tilde{B})\in L(U,A,\tilde{I}\right)$. So, suppose that $\tilde{B}=\left\{\tilde{I}\left(x_{s1},a_{1}\right),\tilde{I}\left(x_{s2},a_{2}\right),\cdots ,\tilde{I}\left(x_{sm},a_{m}\right)\right\}$, where $x_{st}\in U$ $a_{t}\in A$, 1≤s≤|U|, 1≤t≤m and $\tilde{B}(a_{l})=X_{i}^{*}(a_{l})=\tilde{I}\left(x_{sl},a_{l}\right)$. If for any x∈U, $\tilde{I}\left(x,a_{l}\right)\geq \tilde{I}\left(x_{sl},a_{l}\right)$, then let ${\tilde{B}}^{\prime }=\{\tilde{I}\left(x_{s1},a_{1}\right),\tilde{I}\left(x_{s2},a_{2}\right),\cdots ,\tilde{I}\left(x_{sl-1},a_{l-1}\right),$ $\tilde{I}\left(x_{sl+1},a_{l+1}\right),\cdots ,\tilde{I}\left(x_{sm},a_{m}\right)\}$, so we can get $\tilde{B}\prime^{{*}_{D}}={\tilde{B}}^{*}$. □

Otherwise, there exists $x_{k}$, such that $\tilde{I}\left(x_{sl},a_{l}\right)>\tilde{I}\left(x_{k},a_{l}\right)$, then $\tilde{I}\left(x_{sl},a_{l-1}\right)>\tilde{I}\left(x_{k},a_{l-1}\right)$. Denote $\{x_{t1},x_{t2},\cdots ,x_{t\alpha }\}\subseteq U-X_{i}$ to be the set whose elements satisfy the condition that $\tilde{I}\left(x_{t\beta },a_{l}\right)\geq \tilde{I}\left(x_{sl},a_{l}\right)$ (1≤β≤α). Subsequently, $\tilde{I}\left(x_{t\beta },a_{l}\right)>\tilde{I}\left(x_{k},a_{l}\right)$ and thus $\tilde{I}\left(x_{t\beta },a_{l-1}\right)>\tilde{I}\left(x_{k},a_{l-1}\right)$. Let ${\tilde{B}}^{\prime }={\tilde{B}}_{1}\cup {\tilde{B}}_{2}$, where

$$\begin{array}{l} {\tilde{B}}_{1}=\{\tilde{I}\left(x_{s1},a_{1}\right),\tilde{I}\left(x_{s2},a_{2}\right),\cdots ,\tilde{I}\left(x_{sl-1},a_{l-1}\right),\tilde{I}\left(x_{sl+1},a_{l+1}\right),\cdots ,\tilde{I}\left(x_{sm},a_{m}\right)\},\\ {\tilde{B}}_{2}=\{\tilde{I}\left(x_{s1},a_{1}\right),\tilde{I}\left(x_{s2},a_{2}\right),\cdots ,\tilde{I}\left(x_{sl-2},a_{l-2}\right),\underset{1\leq \beta \leq \alpha }{\wedge }\tilde{I}(x_{t\beta },a_{l-1}),\tilde{I}\left(x_{sl+1},a_{l+1}\right),\cdots ,\tilde{I}\left(x_{sm},a_{m}\right)\}, \end{array}$$

then it follows that $\tilde{B}\prime^{{*}_{D}}={\tilde{B}}_{1}{}^{{*}_{D}}\cap {\tilde{B}}_{2}{}^{{*}_{D}}$. Accordingly, we know that, if $x\in {\tilde{B}}^{*}$, i.e., $\forall a_{t}\in A,\tilde{I}\left(x,a_{t}\right)\geq \tilde{I}\left(x_{st},a_{t}\right)$, then $x\in B_{1}^{{*}_{D}}$ and $x\in \{x_{t1},x_{t2},\cdots ,x_{t\alpha }\}$. Afterwards, $x\in {\tilde{B}}_{1}{}^{*}$ and $x\in {\tilde{B}}_{2}{}^{*}$. It follows that $x\in \tilde{B}\prime^{{*}_{D}}$ and so ${\tilde{B}}^{*}\subseteq \tilde{B}\prime^{{*}_{D}}$. If $x\notin {\tilde{B}}^{*}$, then $x\notin B_{1}^{{*}_{D}}$ or $\tilde{I}\left(x_{sl},a_{l}\right)>\tilde{I}\left(x,a_{l}\right)$. If $x\notin B_{1}^{{*}_{D}}$, then $x\in \tilde{B}\prime^{{*}_{D}}$. If $\tilde{I}\left(x_{sl},a_{l}\right)>\tilde{I}\left(x,a_{l}\right)$, then $\tilde{I}\left(x_{t\beta },a_{l-1}\right)>\tilde{I}\left(x,a_{l-1}\right)$, and then $\tilde{I}\left(x_{sl},a_{q\sigma }\right)>\tilde{I}\left(x,a_{q\sigma }\right)$, i.e., $x\notin {\tilde{B}}_{2}{}^{{*}_{D}}$ and so $\underset{1\leq \beta \leq \alpha }{\wedge }\tilde{I}(x_{t\beta },a_{l-1})>\tilde{I}(x,a_{l-1})$ i.e., $x\notin B_{2}^{{*}_{D}}$. Hence, $x\notin \tilde{B}\prime^{{*}_{D}}$. Thus, $\tilde{B}\prime^{{*}_{D}}={\tilde{B}}^{*}$.

Therefore, we conclude that for any $X_{i}\in L_{U}^{f}(U,A,\tilde{I})$, there exists ${\tilde{B}}^{\prime }\in U^{D}$, such that $\tilde{B}\prime^{{*}_{D}}=X_{i}$. In conclusion, $a_{l}$ is an unnecessary attribute.

Moreover, suppose that $a_{l-1}$ is an absolutely necessary attribute and D is a consistent set that contains $a_{l-1}$. Since $a_{l-1}$ is an absolutely necessary attribute, we have $a_{l-1}\in D$, thus $D-\{a_{l}\}$ is also a consistent set, i.e., D is not a reduction. Therefore, $a_{l}$ is an absolutely unnecessary attribute.

**Corollary** **6.** *Let* $\left(U,A,\tilde{I},T,\tilde{J}\right)$ *be an IF generalized consistent decision formal context and* f *:* $L(U,T,\tilde{J})\to L(U,A,\tilde{I})$ *is an implication mapping. Subsequently, for* f *:* a∈A *is an absolutely unnecessary attribute if for any* $X_{i}\in L_{U}^{f}(U,A,\tilde{I})$ *and* $x_{j}\in U-X_{i}$ *,* $X_{i}^{*}(a)>\tilde{I}(x_{j},a)$ *holds.*

### 3.3. Approach to Attribute Reduction in View of the Implication Mapping

The discernibility matrix and discernibility function are useful tools in computing all reductions for information tables [5], which we introduce to compute all reductions for an IF generalized consistent decision formal context that is based on the conclusions discussed above. Furthermore, we discuss the approach to reduction as well as the corresponding characteristics in the following.

**Definition** **18.** *Let*$\left(U,A,\tilde{I},T,\tilde{J}\right)$*be an IF generalized consistent decision formal context,*f*:*$L(U,T,\tilde{J})\to L(U,A,\tilde{I})$*is an implication mapping, and*$X_{i},X_{j}\in L_{U}^{f}(U,A,\tilde{I})\cup \{\{x_{j}\}|x_{j}\in U\}$*, we define*$D_{f}^{*}(X_{i},X_{j})=\{\begin{array}{c} \left\{a\in A|\mu_{X_{i}^{{*}_{A}}}(a)>\mu_{X_{j}^{{*}_{A}}}(a),\gamma_{X_{i}^{{*}_{A}}}(a)<\gamma_{X_{j}^{{*}_{A}}}(a)\right\},X_{i}\in L_{U}^{f}(U,A,\tilde{I}),\;X_{j}=\{x_{j}\}⊄X_{i}\\ \emptyset \;otherwise \end{array},$$D_{f}^{*}={\left(D_{f}^{*}(X_{i},X_{j})\right)}_{|L_{U}^{f}(U,A,\tilde{I})\cup \left\{\left\{x_{j}\right\}|x_{j}\in U\right\}{|}^{2}}$.

Subsequently, $D_{f}^{*}(X_{i},X_{j})$ is called discernibility attributes set between $X_{i}$ and $X_{j}$ based on f. $D^{*}$ is referred as discernibility matrix of an IF formal context based on f.

**Proposition** **17.** *Let $\left(U,A,\tilde{I},T,\tilde{J}\right)$ be an IF generalized consistent decision formal context and f: $L(U,T,\tilde{J})\to L(U,A,\tilde{I})$ is an implication mapping. Afterwards, for D⊆A, the following two propositions are equivalent.*

(1) *D is a consistent set of $\left(U,A,\tilde{I},T,\tilde{J}\right)$ based on f.*
(2) If $D_{f}^{*}(X_{i},X_{j})\neq \emptyset$, then $D\cap D_{f}^{*}(X_{i},X_{j})\neq \emptyset$, $\forall D_{f}^{*}(X_{i},X_{j})\in D_{f}^{*}$.

**Proof.**  (1)⇒(2) We assume that property (2) does not hold. i.e., $\exists X_{i}\in L_{U}^{f}(U,A,\tilde{I})$, $X_{j}=\{x_{j}\}⊄X_{i}$ such that $D_{f}^{*}(X_{i},X_{j})\neq \emptyset \in D_{f}^{*}$ and $D\cap D_{f}^{*}(X_{i},X_{j})=\emptyset$. That is to say ∀a∈D such that $\mu_{X_{i}^{{*}_{A}}}(a)\leq \mu_{X_{j}^{{*}_{A}}}(a)$ and $\gamma_{X_{i}^{{*}_{A}}}(a)\geq \gamma_{X_{j}^{{*}_{A}}}(a)$, hence $x_{j}\in X_{i}^{{*}_{D}{*}_{D}}$. In other words, $\left(X_{i},X_{{}_{i}}^{{*}_{D}}\right)\notin$ $L(U,D,{\tilde{I}}_{D})$. It is paradoxical that D is a consistent set of $\left(U,A,\tilde{I},T,\tilde{J}\right)$ based on f.

(2)⇒(1) If $\forall D_{f}^{*}(X_{i},X_{j})\neq \emptyset \in D_{f}^{*}$, then $\forall a\in D\cap D_{f}^{*}\left(X_{i},X_{j}\right)\neq \emptyset$, such that $\mu_{X_{i}^{{*}_{A}}}(a)>\mu_{X_{j}^{{*}_{A}}}(a)$ or $\gamma_{X_{i}^{{*}_{A}}}(a)<\gamma_{X_{j}^{{*}_{A}}}(a)$ ⇒ $\mu_{X_{i}^{{*}_{D}}}(a)>\mu_{X_{j}^{{*}_{D}}}(a)$ or $\gamma_{X_{i}^{{*}_{D}}}(a)<\gamma_{X_{j}^{{*}_{D}}}(a)$. Hence, $\forall X_{i}\in L_{U}(U,T,\tilde{J})$ and $X_{j}=\{x_{j}\}⊄X_{i}$, such that $x_{j}\notin X_{i}^{{*}_{D}{*}_{D}}\Rightarrow X_{i}=X_{i}^{{*}_{D}{*}_{D}}$. Accordingly, there exists $\left(X_{i},X_{i}^{{*}_{D}})\in L(U,D,{\tilde{I}}_{D}\right)$. Thus, D is a consistent set of $\left(U,A,\tilde{I},T,\tilde{J}\right)$ based on f. □

**Definition** **19.** *Let* $\left(U,A,\tilde{I},T,\tilde{J}\right)$ *be an IF generalized consistent decision formal context,* f *:* $L(U,T,\tilde{J})\to L(U,A,\tilde{I})$ *is an implication mapping and* $D_{f}^{*}$ *is discernibility matrix of* $\left(U,A,\tilde{I},T,\tilde{J}\right)$ *. We define*

$$M_{f}^{*}=\underset{D_{f}^{*}\left(X_{i},X_{j}\right)\in D_{f}^{*}}{\wedge }\{\vee \left\{a_{k}|a_{k}\in D_{f}^{*}\left(X_{i},X_{j}\right)\right\}\},\;D_{f}^{*}(X_{i},X_{j})\neq \emptyset .$$

Subsequently, $M_{f}^{*}$ is called discernibility function of an IF generalized consistent decision formal context based on f.

**Proposition** **18.** *Let* $\left(U,A,\tilde{I},T,\tilde{J}\right)$ *be an IF generalized consistent decision formal context and* f *:* $L(U,T,\tilde{J})\to L(U,A,\tilde{I})$ *is an implication mapping. The minimal disjunctive normal form of discernibility function is*

$$M_{f}^{*}=\overset{p}{\underset{k=1}{\vee }}(\overset{q_{k}}{\underset{s=1}{\wedge a_{s}}}),$$

*Denote $B_{k}^{f_{i}}=\{a_{s}|s\leq q_{k}\}$, then $\left\{B_{k}^{f_{i}}|k\leq p\right\}$ are all reductions of IF generalized consistent decision formal context $\left(U,A,\tilde{I},T,\tilde{J}\right)$, based on f.*

**Proof.**  It can be easily verified by the Proposition 19, Proposition 20, and the definition of minimal disjunctive normal of discernibility function.

In view of the implication f, from the above discussion, we know that to get the attribute reductions in concept lattices based on IF generalized consistent decision formal context, is equal to find the minimum consistent set D, which satisfies $D\cap D_{f}^{*}(X_{i},X_{j})\neq \emptyset$ for any $D_{f}^{*}(X_{i},X_{j})\neq \emptyset$. □

**Corollary** **7.** *Let* $\left(U,A,\tilde{I},T,\tilde{J}\right)$ *be an IF generalized consistent decision formal context and* f *:* $L(U,T,\tilde{J})\to L(U,A,\tilde{I})$ *is an implication mapping.* ∀a∈A *,* a *is the core attribute* ⇔ ∃ $X_{i},X_{j}\in$ $L_{U}^{f}(U,A,\tilde{I})\cup \{\{x_{j}\}|x_{j}\in U\}$ *, such that* $D_{f}^{*}(X_{i},X_{j})=\{a\}$ *.*

**Example** **2.** *(Renewal Example 1) All of the reductions can be computed by discernibility matrix and discernibility function in the Example 1.*

By the definition of discernibility matrix, the results are presented in Table 2.

Hence, we can get that

$$\begin{array}{l} M^{*}=(b\vee c)\wedge (a\vee c\vee d\vee e)\wedge (b\vee e)\wedge (a\vee b\vee c)\wedge (a\vee d\vee e)\wedge d\wedge (d\vee e)\\ =(b\vee e)\vee (b\vee c)\vee d=(b\wedge d)\vee (c\wedge d\wedge e) \end{array}$$

Through calculation and analysis, there are two reductions, which are $D^{1}=\left\{c,b,e\right\}$, $D^{2}=\left\{b,d\right\}$ for the IF formal context in Table 1. a,b,c,d,e are relatively necessary attributes. There are no absolutely unnecessary attribute and absolutely unnecessary attributes in this IF formal context.

## 4. The Reduction of IF Generalized Consistent Decision Formal Context

**Definition** **20.** *Let*$\left(U,A,\tilde{I},T,\tilde{J}\right)$*be an IF consistent generalized decision formal context,*D⊆A*. We say that*D*is a generalized consistent set of*$\left(U,A,\tilde{I},T,\tilde{J}\right)$*, if*$\left(U,D,{\tilde{I}}_{D},T,\tilde{J}\right)$*is generalized consistent. Furthermore, if*D*is a consistent set, and for any*d∈D*,*$\left(U,D-\{d\},{\tilde{I}}_{D-\{d\}},T,\tilde{J}\right)$*is not generalized consistent, then*D*is called an attribute reduction of*$\left(U,A,\tilde{I},T,\tilde{J}\right)$*. The intersection set of all reductions is called the core of*$\left(U,A,\tilde{I},T,\tilde{J}\right)$.

According to above definition, we can obtain the following proposition.

**Proposition** **19.** *Let $\left(U,A,\tilde{I},T,\tilde{J}\right)$ be an IF generalized consistent decision formal context, D⊆A, D≠∅. The following propositions are equivalent.*

(1) *D is a consistent set of $\left(U,A,\tilde{I},T,\tilde{J}\right)$;*
(2) *$L(U,A,\tilde{I})≼L(U,D,{\tilde{I}}_{D}))≼L(U,T,\tilde{J})$;*
(3) *There exists an implication mapping f: $L(U,T,\tilde{J})\to L(U,D,{\tilde{I}}_{D})$*
(4) *$\exists L^{\prime }\subseteq L_{U}(U,D,{\tilde{I}}_{D}),L^{\prime }⋐L_{U}(U,T,\tilde{J}).$*

**Proposition** **20.** *Let* $\left(U,A,\tilde{I},T,\tilde{J}\right)$ *be an IF generalized consistent decision formal context, and a reduction of it must exist.*

**Proof.**  It is similar to the Proposition 12. □

**Corollary** **8.** *Let* $\left(U,A,\tilde{I},T,\tilde{J}\right)$ *be an IF generalized consistent decision formal context. The core is the reduction* ⇔ *The reduction is only one.*

**Corollary** **9.** *Let* $\left(U,A,\tilde{I},T,\tilde{J}\right)$ *be an IF generalized consistent decision formal context.* a∈A *is one core attribute* ⇔A-{a} *is not consistent set.*

**Corollary** **10.** *Let*$\left(U,A,\tilde{I},T,\tilde{J}\right)$*be an IF generalized consistent decision formal context*. a∈A*is unnecessary attribute*⇔A-{a}*is consistent set.*

**Proposition** **21.** *Let*$\left(U,A,\tilde{I},T,\tilde{J}\right)$*be an IF generalized consistent decision formal context. For any*D⊆A*, if there exists implication mapping*$g:L(U,T,\tilde{J})\to L(U,D,{\tilde{I}}_{D})$*in*$\left(U,D,{\tilde{I}}_{D},T,\tilde{J}\right)$*, then there exists implication mapping*$f:L(U,T,\tilde{J})\to L(U,A,\tilde{I})$*, such that*$L_{U}^{f}(U,A,\tilde{I})=L_{U}^{g}(U,D,{\tilde{I}}_{D})$*in*$\left(U,A,\tilde{I},T,\tilde{J}\right)$.

**Proof.**  Since g is an implication mapping, then $L_{U}^{g}(U,D,{\tilde{I}}_{D})\subseteq L(U,D,{\tilde{I}}_{D})$. In view of $L_{U}(U,D,{\tilde{I}}_{D})\subseteq L_{U}(U,A,\tilde{I})$, then $L_{U}^{g}(U,D,{\tilde{I}}_{D})\subseteq L_{U}(U,A,\tilde{I})$. It follows that there exists implication mapping $f:L(U,T,\tilde{J})\to L(U,A,\tilde{I})$, such that $\forall (X,\tilde{B})\in L(U,T,\tilde{J})$, and if $g((X,\tilde{B}))=(X^{\prime },{\tilde{B}}^{\prime })$, then $f((X,\tilde{B}))=(X^{\prime },{\tilde{B}}^{\prime \prime })$. Hence, $L_{U}^{f}(U,A,\tilde{I})=L_{U}^{g}(U,D,{\tilde{I}}_{D})$.

This proposition illustrates that the pruning of the attributes A will not contribute new implication mapping, which is to say that, for any D⊆A, any implication mapping in $\left(U,D,{\tilde{I}}_{D},T,\tilde{J}\right)$ must come from $\left(U,A,\tilde{I},T,\tilde{J}\right)$**,** i.e., $L_{U}^{f}(U,A,\tilde{I})=L_{U}^{g}(U,D,{\tilde{I}}_{D})$. We can conclude that $\left(U,D,{\tilde{I}}_{D},T,\tilde{J}\right)$ is not generalized consistent if $\left(U,A,\tilde{I},T,\tilde{J}\right)$ is not generalized consistent. □

**Proposition** **22.** *Let*$\left(U,A,\tilde{I},T,\tilde{J}\right)$*be an IF generalized consistent decision formal context. The set of all consistent sets of*$\left(U,A,\tilde{I},T,\tilde{J}\right)$*is equal to the set of all the consistent sets based on all implication mappings from*$L(U,T,\tilde{J})$*to*$L(U,A,\tilde{I})$.

**Proof.**  Firstly, let D⊆A be a consistent set of $\left(U,A,\tilde{I},T,\tilde{J}\right)$, then there exists implication mapping $g:L(U,T,\tilde{J})\to L(U,D,{\tilde{I}}_{D})$. So, there must exist $f:L(U,T,\tilde{J})\to L(U,A,\tilde{I})$ such that $L_{U}^{g}(U,D,{\tilde{I}}_{D})=L_{U}^{f}(U,A,\tilde{I})$. $L_{U}^{f}(U,A,\tilde{I})\subseteq L_{U}(U,D,{\tilde{I}}_{D})$, since $L_{U}^{g}(U,D,{\tilde{I}}_{D})\subseteq$ $L_{U}(U,D,{\tilde{I}}_{D})$. Hence, D is the consistent set that is based on f.

Secondly, let $f:L(U,T,\tilde{J})\to L(U,A,\tilde{I})$ be an implication mapping and D is the consistent set based on f, and then $L_{U}^{f}(U,A,\tilde{I})\subseteq L_{U}(U,D,{\tilde{I}}_{D})$, $L_{U}^{f}(U,A,\tilde{I})⋐L_{U}(U,T,J)$. Hence, D is the set of all consistent sets in $\left(U,A,\tilde{I},T,\tilde{J}\right)$. □

**Proposition** **23.** *Let*$\left(U,A,\tilde{I},T,\tilde{J}\right)$*be an IF generalized consistent decision formal context. The set of all reductions of*$\left(U,A,\tilde{I},T,\tilde{J}\right)$*is equal to the set of minimal elements of all reductions that are based on all implication mappings from*$L(U,T,\tilde{J})$*to*$L(U,A,\tilde{I})$.

**Proof.**  Firstly, assume that D⊆A is a reduction of $\left(U,A,\tilde{I},T,\tilde{J}\right)$, then there exists an implication mapping $f:L(U,T,\tilde{J})\to L(U,A,\tilde{I})$, and D is the consistent set based on f. Since for any d∈D, $\left(U,D-\{d\},{\tilde{I}}_{D-\{d\}},T,\tilde{J}\right)$ is not generalized consistent, then $L_{U}^{f}(U,A,\tilde{I})\subseteq$ $L_{U}(U,D-\{d\},{\tilde{I}}_{D-\{d\}})$ does not hold. Thus, D is the reduction based on f. Assume that D is not the minimal element, then there exists another implication mapping $g:L(U,T,\tilde{J})\to L(U,A,\tilde{I})$ and $D^{\prime }\subset D$ is the reduction based on g. It follows that D is not its reduction, which is in clear contradiction with the known condition. Therefore, D is one minimal element of all reductions that are based on all implication mappings from $L(U,T,\tilde{J})$ to $L(U,A,\tilde{I})$.

Secondly, let D⊆A be one minimal element of all reductions based on all implication mappings, then D is the consistent set of $\left(U,A,\tilde{I},T,\tilde{J}\right)$, since D is the consistent set based on one implication mapping. When assuming that D is not the reduction of $\left(U,A,\tilde{I},T,\tilde{J}\right)$, it follows that there exists d∈D, such that $\left(U,D-\{d\},{\tilde{I}}_{D-\{d\}},T,\tilde{J}\right)$ is generalized consistent. Accordingly, implication mapping exists $f:L(U,T,\tilde{J})\to L(U,A,\tilde{I})$, such that D−{d} is the consistent set of $\left(U,A,\tilde{I},T,\tilde{J}\right)$ based on f. Thus, there must exist $D^{\prime }\subset D$, such that is the reduction of $\left(U,A,\tilde{I},T,\tilde{J}\right)$ based on f, which clearly contradict the known condition that D is one minimal element. Therefore, D is the reduction of $\left(U,A,\tilde{I},T,\tilde{J}\right)$.

According to this proposition, we can find that, to find the reductions of one IF generalized consistent decision formal context is equal to finding all reductions based on all implication mappings. In the view of Proposition 10, we conclude that it only needs to find the reductions based on any implication mapping for which have the same range. □

**Corollary** **11.** *Let $\left(U,A,\tilde{I},T,\tilde{J}\right)$ be an IF generalized consistent decision formal context. Subsequently, all reductions of $\left(U,A,\tilde{I},T,\tilde{J}\right)$ can be defined as*

$$M^{*}=\overset{t}{\underset{f}{\vee }}M_{f}^{*}=\overset{p}{\underset{k=1}{\vee }}(\overset{q_{k}}{\underset{s=1}{\wedge a_{s}}}),\;(\mathrm{where}\;f\;\text{varies from all implication mappings}).$$

*Denote $B_{k}=\left\{a_{s}|s=1,2,\cdots q_{k}\right\}$, then $\left\{B_{k}|k=1,2,\cdots p\right\}$ are all reductions of IF generalized consistent decision formal context $\left(U,A,\tilde{I},T,\tilde{J}\right)$.*

**Example** **3.** *(Renewal Example 1.) According to Corollary 11, we can ascertain all of the reductions of IF generalized consistent decision formal context that are displayed in Table 1. We classify all implication mappings according to their ranges and all the possible ranges. Reductions are shown, as follows;*

(1) $\left\{(1,{\tilde{A}}_{1}\right)$, $\left(2,{\tilde{A}}_{2}\right)$, $\left(4,{\tilde{A}}_{3}\right)$, $\left(12,{\tilde{A}}_{4}\right)$, $\left(24,{\tilde{A}}_{7}\right)$, $\left(U,{\tilde{A}}_{10}\right)$, $\left(\emptyset ,\tilde{A})\right\}$
$\begin{array}{l} M_{f}^{*}=(b\vee c)\wedge (a\vee c\vee d\vee e))\wedge (b\vee e)\wedge (a\vee b\vee c)\wedge (a\vee d\vee e)\\ =(b\vee c)\wedge (b\vee e)\wedge (a\vee d\vee e)=(a\wedge b)\vee (b\wedge d)\vee (b\wedge e)\vee (c\wedge e) \end{array}$
(2) $\left\{(1,{\tilde{A}}_{1}\right)$, $\left(2,{\tilde{A}}_{2}\right)$, $\left(4,{\tilde{A}}_{3}\right)$, $\left(13,{\tilde{A}}_{5}\right)$, $\left(24,{\tilde{A}}_{7}\right)$, $\left(U,{\tilde{A}}_{10}\right)$, $\left(\emptyset ,\tilde{A})\right\}$
$\begin{array}{l} M_{f}^{*}=(b\vee c)\wedge (a\vee c\vee d\vee e)\wedge (b\vee e)\wedge (a\vee b\vee c)\wedge (a\vee d\vee e)\wedge d\wedge (d\vee e)\\ =(b\vee e)\vee (b\vee c)\vee d=(b\wedge d)\vee (c\wedge d\wedge e) \end{array}$
(3) $\left\{(1,{\tilde{A}}_{1}\right)$, $\left(2,{\tilde{A}}_{2}\right)$, $\left(4,{\tilde{A}}_{3}\right)$, $\left(14,{\tilde{A}}_{6}\right)$, $\left(24,{\tilde{A}}_{7}\right)$, $\left(U,{\tilde{A}}_{10}\right)$, $\left(\emptyset ,\tilde{A})\right\}$
$\begin{array}{l} M_{f}^{*}=(b\vee c)\wedge (a\vee c\vee d\vee e)\wedge (b\vee e)\wedge (a\vee b\vee c)\wedge (a\vee d\vee e)\wedge (a\vee b\vee e)\wedge e\\ =(b\vee c)\wedge e=(b\wedge e)\vee (c\wedge e) \end{array}$
(4) $\left\{(1,{\tilde{A}}_{1}\right)$, $\left(2,{\tilde{A}}_{2}\right)$, $\left(4,{\tilde{A}}_{3}\right)$, $\left(24,{\tilde{A}}_{7}\right)$, $\left(124,{\tilde{A}}_{9}\right)$, $\left(U,{\tilde{A}}_{10}\right)$, $\left(\emptyset ,\tilde{A})\right\}$
$\begin{array}{l} M_{f}^{*}=(b\vee c)\wedge (a\vee c\vee d\vee e)\wedge (b\vee e)\wedge (a\vee b\vee c)\wedge (a\vee d\vee e)\\ =(b\vee c)\wedge (b\vee e)\wedge (a\vee d\vee e)=(a\wedge b)\vee (b\wedge d)\vee (b\wedge e)\vee (c\wedge e) \end{array}$
(5) $\left\{(2,{\tilde{A}}_{2}\right)$, $\left(4,{\tilde{A}}_{3}\right)$, $\left(12,{\tilde{A}}_{4}\right)$, $\left(13,{\tilde{A}}_{5}\right)$, $\left(24,{\tilde{A}}_{7}\right)$, $\left(U,{\tilde{A}}_{10}\right)$, $\left(\emptyset ,\tilde{A})\right\}$
$\begin{array}{l} M_{f}^{*}=(b\vee c)\wedge (a\vee c\vee d\vee e)\wedge (b\vee e)\wedge (a\vee b\vee c)\wedge (a\vee d\vee e)\wedge d\wedge (d\vee e)\\ =(b\vee e)\vee (b\vee c)\vee d=(b\wedge d)\vee (c\wedge d\wedge e) \end{array}$
(6) $\left\{(2,{\tilde{A}}_{2}\right)$, $\left(4,{\tilde{A}}_{3}\right)$, $\left(12,{\tilde{A}}_{4}\right)$, $\left(14,{\tilde{A}}_{6}\right)$, $\left(24,{\tilde{A}}_{7}\right)$, $\left(U,{\tilde{A}}_{10}\right)$, $\left(\emptyset ,\tilde{A})\right\}$
$\begin{array}{l} M_{f}^{*}=(b\vee c)\wedge (a\vee c\vee d\vee e)\wedge (b\vee e)\wedge (a\vee b\vee c)\wedge (a\vee d\vee e)\wedge (a\vee b\vee e)\wedge e\\ =(b\vee c)\wedge e=(b\wedge e)\vee (c\wedge e) \end{array}$
(7) $\left\{(2,{\tilde{A}}_{2}\right)$, $\left(4,{\tilde{A}}_{3}\right)$, $\left(13,{\tilde{A}}_{5}\right)$, $\left(14,{\tilde{A}}_{6}\right)$, $\left(24,{\tilde{A}}_{7}\right)$, $\left(U,{\tilde{A}}_{10}\right)$, $\left(\emptyset ,\tilde{A})\right\}$
$\begin{array}{l} M_{f}^{*}=(b\vee c)\wedge (a\vee c\vee d\vee e)\wedge (b\vee e)\wedge (a\vee b\vee c)\wedge e\wedge (a\vee b\vee e)\wedge d\wedge (d\vee e)\\ =(b\vee c)\wedge e\wedge d=(b\wedge d\wedge e)\vee (c\wedge d\wedge e) \end{array}$
(8) $\left\{(2,{\tilde{A}}_{2}\right)$, $\left(4,{\tilde{A}}_{3}\right)$, $\left(13,{\tilde{A}}_{5}\right)$, $\left(24,{\tilde{A}}_{7}\right)$, $\left(124,{\tilde{A}}_{9}\right)$, $\left(U,{\tilde{A}}_{10}\right)$, $\left(\emptyset ,\tilde{A})\right\}$
$\begin{array}{l} M_{f}^{*}=(b\vee c)\wedge (a\vee c\vee d\vee e)\wedge (b\vee e)\wedge (a\vee b\vee c)\wedge d\wedge (d\vee e)\wedge (a\vee b\vee c)\\ =(b\vee e)\vee (b\vee c)\vee d=(b\wedge d)\vee (c\wedge d\wedge e) \end{array}$
(9) $\left\{(2,{\tilde{A}}_{2}\right)$, $\left(4,{\tilde{A}}_{3}\right)$, $\left(14,{\tilde{A}}_{6}\right)$, $\left(24,{\tilde{A}}_{7}\right)$, $\left(124,{\tilde{A}}_{9}\right)$, $\left(U,{\tilde{A}}_{10}\right)$, $\left(\emptyset ,\tilde{A})\right\}$
$\begin{array}{l} M_{f}^{*}=(b\vee c)\wedge (a\vee c\vee d\vee e)\wedge (b\vee e)\wedge (a\vee b\vee c)\wedge (a\vee d\vee e)\wedge (a\vee b\vee e)\wedge e\\ =(b\vee c)\wedge e=(b\wedge e)\vee (c\wedge e) \end{array}$

Furthermore, all reductions of $\left(U,A,\tilde{I},T,\tilde{J}\right)$ are

$$M^{*}=\overset{t}{\underset{f}{\vee }}M_{f}^{*}=\overset{p}{\underset{k=1}{\vee }}\left(\overset{q_{k}}{\underset{s=1}{\wedge a_{s}}}\right)=(a\wedge b)\vee (b\wedge e)\vee (b\wedge d)\vee (c\wedge e)$$

Conclusively, the reductions of the IF generalized consistent decision formal context are {a,b}, {b,e}, {b,d}, and {c,e}, respectively. All of the attributes are, respectively, necessary attributes and there are no absolutely unnecessary attributes and absolutely necessary attributes for this formal context.

## 5. Algorithm and Case Study of Data Analysis in Intuitionistic Fuzzy Generalized Consistent Decision Formal Context

**Figure 5:**
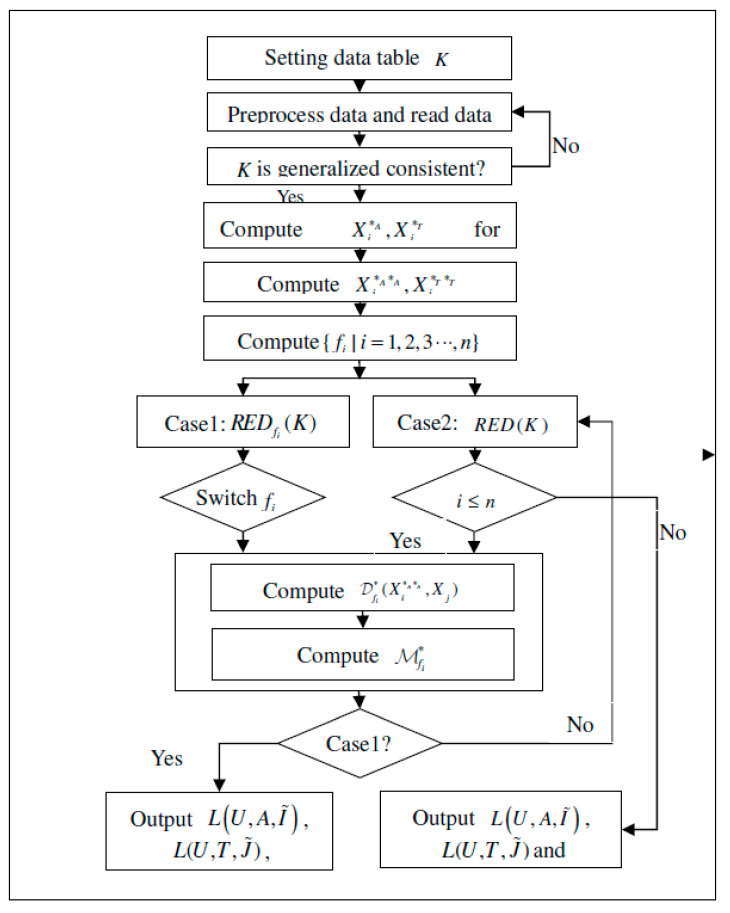
The flow chart of the Algorithm 5.1.

Experimental computing program can be designed and carried out so as to apply the algorithm that is studied more directly in an applicable manner. The main process of the program will be introduced by the flow chart. According to Algorithm 1, the process of the program can be designed and listed in the following Figure (Figure 5): The flow chart of the program.

Algorithm 1 Discernibility functions are monotonic Boolean functions and we obtain that the normal minimal disjunctive form of the discernibility function determined all of the reductions. Algorithm of concept lattice and attribute reduction in the IF generalized consistent decision formal context is described, as follows, and the flow chart of the Algorithm is shown in Figure 3.

**Algorithm 1** Data Analysis in Intuitionistic Fuzzy Generalized Consistent Decision Formal Context**Input:** An IF decision formal context $K=(U,A,\tilde{I},T,\tilde{J})$, where $U=\{x_{1},x_{2},\cdots ,x_{n}\}$, $A=\{a_{1},a_{2},\cdots ,a_{m}\}$, and $T=\{t_{1},t_{2},\cdots ,t_{m}\}$.**Output:**$L(U,A,\tilde{I},)$, $L(U,T,\tilde{J})$, $\left\{RED_{f_{i}}(K)|i=1,2,3\cdots n\right\}$ or RED(K) // All concepts of $\left(U,A,\tilde{I},\right)$ and $\left(U,T,\tilde{J}\right)$, reductions based on $\left\{f_{i}|i=1,2,3\cdots ,n\right\}$, and reductions of K.*Step 1*: Initialized setting. We denote the initialized target information system by K, and read data table and preprocess data. *Step 2*: Judge whether K is generalized consistent or not. If K is generalized consistent, go to *Step3*; else go back to *Step 1.**Step 3*: Compute $X_{i}^{{*}_{A}},X_{i}^{{*}_{T}}$ for $X_{i}\in P(U)$.*Step 4*: Compute $X_{i}^{{*}_{A}{*}_{A}},X_{i}^{{*}_{T}{*}_{T}}$, the corresponding extension of $X_{i}^{{*}_{A}},X_{i}^{{*}_{T}}$, according to the Definition 6 and Definition 7. Remove the repetitive.*Step 5*: Compute all implication mappings $\left\{f_{i}|i=1,2,3\cdots ,n\right\}$ i.e., $\left\{L_{U}^{f_{i}}(U,A,\tilde{I},)|i\leq n\right\}$.*Step 6:* Switch. Case1: compute $RED_{f_{i}}(K)(i=1,2,3\cdots n)$ go to *Step 7*, Case 2: compute RED(K), go to *Step 8.**Step 7:* Chose one implication mapping $f_{i}$, go to *Step 9.**Step 8:* If i≤n, go to *Step 9*, else go to *Step 13*. *Step 9*: Compute the set of discernibility attributes $D_{f_{i}}^{*}(X_{i}^{{*}_{A}{*}_{A}},X_{j})$, according to Definition 18*Step10*: Compute $M_{f_{i}}^{*}=\underset{D_{f_{i}}^{*}((X_{i}^{{*}_{A}{*}_{A}},X_{j})\in D_{f_{i}}^{*}}{\wedge }\left\{\vee \left\{a_{k}|a_{k}\in D_{f_{i}}^{*}(X_{i}^{{*}_{A}{*}_{A}},X_{j})\right\}\right\}$.*Step 11*: Compute $M_{f_{i}}^{*}=\overset{p}{\underset{k=1}{\vee }}\left(\overset{q_{k}}{\underset{s=1}{\wedge a_{s}}}\right)$.*Step 12*: If case 1, let $B_{k}^{f_{i}}=\{a_{s}|s\leq q_{k}\}$ and $\left\{B_{k}^{f_{i}}|k\leq p\right\}$ be all reductions of K based on f and go to *Step 14.* Otherwise, go back to *Step 8.**Step 13*: Compute $M^{*}=\overset{n}{\underset{i}{\vee }}M_{f_{i}}^{*}=\overset{p}{\underset{k=1}{\vee }}\left(\overset{q_{k}}{\underset{s=1}{\wedge a_{s}}}\right)$. Let $B_{k}=\left\{a_{s}|s\leq q_{k}\right\}$ and $RED(K)=\left\{B_{k}|k\leq p\right\}$.Jump to *Step 15.**Step 14*: Stop with $L\left(U,A,\tilde{I}\right)$, $L(U,T,\tilde{J})$ and RED(K) as output.*Step 15:* Stop with $L\left(U,A,\tilde{I}\right)$, $L(U,T,\tilde{J})$ and $\left\{RED_{f_{i}}(K)|i=1,2,3\cdots n\right\}$ as output.

In the worst case, the time complexity of the proposed algorithm is $O{\left(2^{\left|U\right|}|U||A|\right)}^{2}$, where |U| is the number of objects and |A| is the number of attributes. If the number of attributes has an upper bound, as usually happens, the cost of time geometrically grows with the number of objects. Although this algorithm has relatively high time complexity and there is a large number of implication mappings, one implication of mapping can mostly satisfied the research demand in the real world. Therefore, we usually chose Case 1 to reach the aim of the research.

Furthermore, the program has been employed to compute all concepts and reductions of the IF formal context in Example 1, which are consistent with the results that are obtained above. The test shows that the program is effective.

**Example** **4.** *Let* $K=(U,A,\tilde{I},T,\tilde{J})$ *be an IF decision formal context about some emerging viruses presented in Table 3, where* $U=\{x_{1},x_{2},x_{3},x_{4},x_{5},x_{6},x_{7},x_{8},x_{9},x_{10}\}$ *is the set of emerging viruses,* $A=\{a_{1},a_{2},a_{3},a_{4},a_{5}\}$ *and* $T=\{d_{1},d_{2},d_{3}\}$ *are the conditional attribute set and decision attribute set of some important characteristics about viruses, respectively. The interpretations of the attributes are listed, as follows:*

*$a_{1}$—The type of genome’s nucleic acids, where membership degree is referred to DNA, non-membership degree is referred to RNA.*

*$a_{2}$—Envelope.*

*$a_{3}$—Strand: membership degree is referred to single strand, non-membership degree is referred to double strand.*

*$a_{4}$—The heredity of protein.*

*$a_{5}$—Greater genetic variability: membership degree is referred to greater genetic variability, non-membership degree is referred to opposite.*

*$d_{1}$—Pathogenicity: non-membership degree is referred to pathogenicity, membership degree is referred to opposite.*

*$d_{2}$—Economic value.*

*$d_{3}$—The value of scientific research.*

There are ninety-four concepts in $L(U,A,\tilde{I})$ and fifteen ones in $L(U,T,\tilde{J})$ by computing. For simplicity, the extensions will be shown in the following Table 4 only, where $\left\{x_{i},\cdots ,x_{h}\right\}$ is denoted by $x_{i,\cdots ,h}$.

We can obtain ninety-four and fifteen concepts, respectively, and one reduction RED(K)=A is obtained, which illustrates that these conditional attributes are all necessary in the IF consistent decision formal context. A detailed description is shown in Table 4.

In fact, there are one thousand and twenty-four possible relationships among the ten viruses in the view of conditional attributes and decision attributes, respectively. By the experimental computing program, we only need to consider ninety-four from $\left(U,A,\tilde{I}\right)$ and fifteen concepts $\left(U,T,\tilde{J}\right)$. Furthermore, concept lattice $L(U,A,\tilde{I})$, $L(U,T,\tilde{J})$ are established, respectively, which clearly provide the layer structure among these objects and are helpful in studying themselves and the relationship between $L(U,A,\tilde{I})$ and $L(U,T,\tilde{J})$. Taking account to the decision attributes, we continue study the reductions based on different implication mapping. Accordingly, in Example 4, pathogenicity, economic value, and the value of scientific research are taken into consideration to analyse the layer structure and reduction of the concepts in $L(U,A,\tilde{I})$. Different implication mapping stands for different preference of decision-maker or decision-making-unit’s over conditional attributes. Researchers can chose to study the partial ones. If taking the implication mapping f:$L(U,T,\tilde{J})\to L(U,A,\tilde{I})$ in the following Table 5, the corresponding reductions are shown in Table 6.

The results that were obtained above presented the relations among these new viruses that may be useful in finding their ancestors and evolution mechanism and the secret of Virus infection principle for the Viruses researchers. It may inspire the investors to put these viruses into commercial application.

## 6. Conclusions

Intuitionistic fuzzy theory and concept lattice theory are two different theories. This paper first combines the intuitionistic fuzzy theory with the concept lattice theory and then proposes one kind of concept lattice in intuitionistic fuzzy generalized consistent decision formal context. In view of the implication mapping, we offered the corresponding definitions and propositions of attribute reduction in concept lattices that were examined by some examples and some propositions to determine the type of attribute investigated. Relative to the classical concept lattice and the fuzzy concept lattice, the concept lattice introducing the intuitionistic fuzzy and implication mapping can obtain more useful information and description to accurately represent the knowledge. However, not all knowledge is useful, and redundant information can interfere with decision makers making the right decisions. So much useless information could be thrown off, since they had much less effect on necessary knowledge representation that they could be ignored by attribute reduction, making the representation of implicit knowledge simpler. What is more, the discernibility matrix and discernibility function in the concept lattice were established and the relation with the reduction based on the implication mappings were investigated, which presented an approach to the attribute reduction of concept lattice based on the intuitionistic fuzzy generalized consistent decision formal context and made it more easy to compute reductions. The experiments were implemented to illustrate the algorithm of data analysis that is designed in this paper. The results of this paper extended the theory of concept lattice and may make great effect on practical applications in the future.

## Figures and Tables

**Table 1:** Intuitionistic fuzzy (IF) decision formal context $\left(U,A,\tilde{I},T,\tilde{J}\right)$.

	a	b	c	d	e	f	g	h
$x_{1}$	<0.9,0.0>	<0.7,0.2>	<0.2,0.5>	<0.9,0.1>	<0.8,0.1>	<0.2,0.8>	<0.1,0.8>	<0.6,0.3>
$x_{2}$	<0.8,0.1>	<0.8,0.2>	<0.8,0.1>	<0.3,0.5>	<0.2,0.7>	<0.9,0.1>	<0.4,0.5>	<0.6,0.2>
$x_{3}$	<0.1,0.8>	<0.7,0.3>	<0.1,0.9>	<0.8,0.2>	<0.2,0.7>	<0.8,0.2>	<0.3,0.7>	<0.5,0.2>
$x_{4}$	<0.7,0.2>	<0.8,0.1>	<0.7,0.1>	<0.2,0.6>	<0.1,0.6>	<0.8,0.1>	<0.7,0.1>	<0.7,0.2>

**Table 2:** Discernibility matrix in Example 1.

$X_{i}/X_{j}$	$x_{1}$	$x_{2}$	$x_{3}$	$x_{4}$	1	2	4	13	24	U	∅
$x_{1}$	∅	ade	A	ade	∅	∅	ade	∅	∅	∅	∅
$x_{2}$	bc	∅	abc	acde	bc	∅	acde	∅	∅	∅	∅
$x_{3}$	∅	∅	∅	∅	∅	∅	∅	∅	∅	∅	∅
$x_{4}$	bc	be	abce	∅	bc	bd	∅	∅	∅	∅	∅
1	∅	ade	A	ade	∅	ade	ade	∅	∅	∅	∅
2	bc	∅	abc	acde	bc	∅	acde	∅	∅	∅	∅
4	bc	be	abce	∅	bc	be	∅	∅	∅	∅	∅
13	∅	d	∅	de	∅	d	de	∅	∅	∅	∅
24	bc	∅	abc	∅	bc	∅	∅	∅	∅	∅	∅
*U*	∅	∅	∅	∅	∅	∅	∅	∅	∅	∅	∅
∅	∅	∅	∅	∅	∅	∅	∅	∅	∅	∅	∅

**Table 3:** The target IF formal context K.

	$a_{1}$	$a_{2}$	$a_{3}$	$a_{4}$	$a_{5}$	$d_{1}$	$d_{2}$	$d_{3}$
$x_{1}$	<0.9,0.0>	<0.8,0.1>	<0.1,0.8>	<0.4,0.5>	<0.1,0.8>	<0.9,0.0>	<0.7,0.0>	<0.0,0.9>
$x_{2}$	<0.8,0.1>	<0.8,0.1>	<0.8,0.1>	<0.3,0.5>	<0.0,0.9>	<0.0,0.8>	<0.7,0.0>	<0.0,0.9>
$x_{3}$	<0.0,0.8>	<0.0,0.6>	<0.0,0.9>	<0.6,0.2>	<0.0,0.8>	<0.0,0.8>	<0.1,0.8>	<0.8,0.0>
$x_{4}$	<0.7,0.2>	<0.8,0.1>	<0.8,0.1>	<0.2,0.6>	<0.8,0.1>	<0.0,0.8>	<0.1,0.8>	<0.3,0.6>
$x_{5}$	<0.0,0.0>	<0.1,0.0>	<0.0,0.0>	<0.9,0.1>	<0.3,0.6>	<0.0,0.8>	<0.9,0.0>	<0.9,0.0>
$x_{6}$	<0.6,0.4>	<0.8,0.0>	<0.0,0.9>	<0.9,0.1>	<0.7,0.2>	<0.0,0.8>	<0.5,0.4>	<0.8,0.0>
$x_{7}$	<0.0,0.4>	<0.1,0.1>	<0.6,0.1>	<0.6,0.1>	<0.1,0.9>	<0.0,0.8>	<0.5,0.4>	<0.8,0.0>
$x_{8}$	<0.0,0.1>	<0.1,0.0>	<0.0,0.0>	<0.9,0.1>	<0.3,0.6>	<0.0,0.8>	<0.9,0.0>	<0.8,0.0>
$x_{9}$	<0.9,0.1>	<0.8,0.0>	<0.8,0.1>	<0.9,0.0>	<0.8,0.1>	<0.3,0.7>	<0.7,0.0>	<0.9,0.0>
$x_{10}$	<0.8,0.1>	<0.7,0.2>	<0.1,0.8>	<0.3,0.5>	<0.3,0.6>	<0.0,0.8>	<0.5,0.4>	<0.3,0.6>

**Table 4:** Extensions of $L(U,A,\tilde{I})$, $L(U,T,\tilde{J})$ and RED(K).

Layer	$L_{U}(U,A,\tilde{I})$	$L_{U}(U,T,\tilde{J})$
0	∅	∅
1	$x_{1}$,$x_{5}$,$x_{9}$	$x_{1}$,$x_{5}$
2	$x_{1,5}$,$x_{1,9}$,$x_{2,9}$,$x_{4,9}$,$x_{5,8}$,$x_{6,9}$,$x_{7,9}$,$x_{9,10}$	$x_{1,5}$,$x_{5,8}$,$x_{5,9}$
3	$x_{1,2,9}$,$x_{1,4,9}$,$x_{1,6,9}$,$x_{1,7,9}$,$x_{1,9,1}$,$x_{2,4,9}$,$x_{2,7,9}$,$x_{4,6,9}$,$x_{4,7,9},x_{4,9,10},x_{5,8,9},x_{6,9,10}$,	$x_{1,5,9}$,$x_{5,8,9}$
4	$x_{1,2,4,9},$ $x_{1,2,6,9},x_{1,2,7,9},$ $x_{1,2,9,10},x_{1,4,6,9},$ $x_{1,4,7,9},$ $x_{1,4,9,10},x_{1,5,8,9},x_{1,6,9,10},x_{1,7,9,10},$ $x_{2,4,7,9},$ $x_{2,5,8,9},$ $x_{4,5,8,9},x_{4,6,9,10},x_{5,6,8,9},$ $x_{5,7,8,9},$ $x_{5,8,9,10}$	-
5	$x_{1,2,4,6,9},x_{1,2,4,7,9},x_{1,2,4,9,10},x_{1,2,5,8,9},x_{1,2,6,9,10},x_{1,2,7,9,10},$ $x_{1,2,4,8,9},$ $x_{1,4,6,9,10},$ $x_{1,4,7,9,10},$ $x_{1,5,6,8,9},x_{1,5,7,8,9},x_{1,5,8,9,10},x_{2,4,5,8,9},x_{2,5,7,8,9},x_{3,5,6,8,9},x_{4,5,6,8,9},x_{4,5,7,8,9},x_{4,5,8,9,10},$ $x_{5,6,7,8,9},$ $x_{5,6,8,9,10}$	$x_{1,2,5,8,9}$,$x_{5,6,7,8,9}$
6	$x_{1,2,4,5,8,9},x_{1,2,4,6,9,10},x_{1,2,4,7,9,10},x_{1,2,5,7,8,9},x_{1,2,5,8,9,10},x_{1,3,5,6,8,9},x_{1,4,5,6,8,9},x_{1,4,5,7,8,9},$ $x_{1,4,5,8,9,10},x_{1,5,6,7,8,9},x_{1,5,6,8,9,10},x_{1,5,7,8,9,10},$ $x_{2,4,5,7,8,9},$ $x_{3,5,6,7,8,9},x_{4,5,6,7,8,9},$	$x_{3,5,6,7,8,9}$,$x_{5,6,7,8,9,10}$
7	$U-x_{3,6,7},U-x_{3,4,10},U-x_{3,4,6},$ $U-x_{2,4,10},$ $U-x_{2,4,7},$ $U-x_{2,3,7},$ $U-x_{2,3,6},$ $U-x_{2,3,4},$	-
8	$U-x_{3,10},U-x_{3,6},U-x_{3,4},U-x_{2,7},U-x_{2,3},$	$U-x_{3,4}$,$U-x_{1,2}$,
9	$U-x_{4},U-x_{3},$	-
10	U	U
Grand total	94	15
RED(K)	A	

**Table 5:** The implication mapping *f*: $L(U,T,\tilde{J})\to L(U,A,\tilde{I})$.

$L(U,T,\tilde{J})$		$x_{1}$	$x_{5}$	$x_{1,5}$	$x_{5,8}$	$x_{5,9}$	$x_{1,5,9}$	$x_{5,8,9}$	$x_{1,2,5,8,9}$	$x_{5,6,7,8,9}$	$x_{3,5,6,7,8,9}$	$x_{5,6,7,8,9,10}$	$U-x_{3,4}$	$U-x_{1,2}$	U
$L(U,A,\tilde{I})$		$x_{1}$	$x_{5}$	$x_{1,5}$	$x_{5,8}$	$x_{9}$	$x_{1,9}$	$x_{5,8,9}$	$x_{2,9}$	$x_{6,9}$	$x_{7,9}$	$x_{9,10}$	$x_{1,6,9}$	$x_{4,9}$	U

**Table 6:** The results of Example 4.

Concept	Extension	Intension	Discernibility Function of *X_i_*
1	$x_{1}$	{<0.9,0.0>,<0.8,0.1>,<0.1,0.8>,<0.4,0.5>,<0.1,0.8>}	$a_{1}$
2	$x_{5}$	{<0.0,0.0>,<0.1,0.0>,<0.0,0.0>,<0.9,0.1>,<0.3,0.6>}	$a_{1}$
3	$x_{1,5}$	{<0.0,0.0>,<0.0,0.1>,<0.0,0.8>,<0.4,0.5>,<0.1,0.8>}	$a_{1}$
4	$x_{5,8}$	{<0.0,0.1>,<0.1,0.0>,<0.0,0.0>,<0.9,0.1>,<0.3,0.6>}	$a_{3}$
5	$x_{9}$	{<0.9,0.1>,<0.8,0.0>,<0.8,0.1>,<0.9,0.0>,<0.8,0.1>}	$\left(a_{1}\wedge a_{2})\vee (a_{1}\wedge a_{3})\vee (a_{2}\wedge a_{3}\right)$ $\vee (a_{1}\wedge a_{5})\vee (a_{2}\wedge a_{5})\vee a_{4}$
6	$x_{19}$	{<0.9,0.1>,<0.8,0.1>,<0.1,0.8>,<0.4,0.5>,<0.1,0.8>}	$a_{1}\vee (a_{3}\wedge a_{4})$
7	$x_{5,8,9}$	{<0.0,0.1>,<0.1,0.0>,<0.0,0.1>,<0.9,0.1>,<0.3,0.6>}	$\left(a_{1}\wedge a_{5})\vee (a_{1}\wedge a_{2})\vee (a_{1}\wedge a_{4}\right)$ $\vee (a_{2}\wedge a_{3})$
8	$x_{2,9}$	{<0.8,0.1>,<0.8,0.1>,<0.8,0.1>,<0.3,0.5>,<0.0,0.9>	$\left(a_{1}\wedge a_{3})\vee (a_{3}\wedge a_{4}\right)$
9	$x_{6,9}$	{<0.6,0.4>,<0.8,0.0>,<0.8,0.1>,<0.9,0.0>,<0.8,0.1>}	$\left(a_{1}\wedge a_{2})\vee (a_{2}\wedge a_{4}\right)$
10	$x_{7,9}$	{<0.0,0.4>,<0.1,0.1>,<0.6,0.1>,<0.6,0.1>,<0.1,0.9>}	$a_{3}\vee a_{4}$
11	$x_{9,10}$	{<0.8,0.1>,<0.7,0.2>,<0.1,0.8>,<0.3,0.5>,<0.4,0.6>}	$\left(a_{1}\wedge a_{5})\vee (a_{3}\wedge a_{4}\wedge a_{5}\right)$
12	$x_{1,6,9}$	{<0.6,0.4>,<0.8,0.1>,<0.0,0.9>,<0.4,0.5>,<0.1,0.8>}	$(a_{1}\wedge a_{4})\vee a_{2}$
13	$x_{4,9}$	{<0.7,0.2>,<0.8,0.1>,<0.1,0.8>,<0.3,0.5>,<0.0,0.9>}	$a_{5}$
14	U	{<0.0,0.8>,<0.0,0.6>,<0.0,0.9>,<0.2,0.6>,<0.0,0.9>}	---
15	∅	{<0.1,0.0>,<0.1,0.0>,<0.1,0.0>,<0.1,0.0>,<0.1,0.0>}	$\vee_{i=1}^{5}a_{i}$
$RED_{f_{i}}(K)$	$\left\{a_{1},a_{3},a_{4},a_{5}\right\}$		
